# Novel intranasal delivery of sihosogansan demonstrates rapid antidepressant activity via GABAergic and BDNF/TrkB pathways: identification of potential bioactive quality markers

**DOI:** 10.1186/s13020-025-01219-6

**Published:** 2025-10-06

**Authors:** Khoa Nguyen Tran, Yeasmin Akter Munni, Ly Thi Huong Nguyen, Tae Woo Oh, Ho Jin Choi, Il Soo Moon, In-Jun Yang

**Affiliations:** 1 Department of Physiology, Dongguk University College of Korean Medicine, Gyeongju, 38066 Republic of Korea; 2 Department of Neuropsychiatry, College of Korean Medicine, Dongguk University, Goyang, 10326 Republic of Korea; 3 Korean Medicine (KM)-Application Center, Korea Institute of Oriental Medicine (KIOM), Daegu, 41062 Republic of Korea; 4 Department of Korean Convergence Medical Science, University of Science & Technology (UST), 1672 Yuseongdae-Ro, Yuseong-Gu, Daejeon, 34054 Republic of Korea; 5 Department of Anatomy, College of Medicine, Dongguk University, Gyeongju, 38066 Republic of Korea; 6 Medical Institute of Dongguk University, Gyeongju, 38066 Republic of Korea

**Keywords:** Sihosogansan, Chaihu-shugan-san, Rapid-acting antidepressant, Intranasal administration, Neuritogenesis, Q-marker

## Abstract

**Background:**

Sihosogansan (SHSGS) is a traditional medicine used to treat depression. However, conventional oral administration requires high doses and prolonged treatment periods. This study aimed to investigate the rapid antidepressant effects of intranasal SHSGS and to identify its Q-markers.

**Methods:**

In zebrafish, SHSGS effects were evaluated in an MK-801-induced anxiety model using electroencephalogram (EEG) recordings. In mice, the rapid effects of intranasal versus oral SHSGS were compared through the open field and tail suspension tests. Mechanistic investigations combined computational network analysis with molecular studies of hippocampal tissue and primary neurons. Q-markers were identified through the integrative analysis of gas chromatography–mass spectrometry data, molecular docking, and experimental validation in behavioral and cellular models.

**Results:**

SHSGS normalized MK-801-induced EEG abnormalities within 30 min in zebrafish, particularly restoring delta/beta and theta/beta ratios. In mice, intranasal SHSGS showed rapid anxiolytic and antidepressant effects at 30 min post-administration, whereas oral administration had no significant effect. SHSGS enhanced gamma-aminobutyric acid (GABA)ergic signaling by increasing hippocampal GABA type B receptor subunit 1, glutamate decarboxylase 67, and GABA levels, while activating the brain-derived neurotrophic factor/tropomyosin receptor kinase B/extracellular signal-regulated protein kinase (BDNF/TrkB/ERK) pathways. Three monoterpenes β-pinene, terpinen-4-ol, and α-terpineol were identified as bioactive Q-markers of SHSGS based on their consistent antidepressant-like effects across behavioral, cellular, and molecular assays. Inhibitor experiments further revealed that α-terpineol’s action required GABA_B1_ receptor signaling, while β-pinene and terpinen-4-ol showed indirect dependency on GABA_B1_ receptor or TrkB pathways.

**Conclusion:**

These findings demonstrate that intranasal SHSGS acts rapidly against depression through the GABAergic and BDNF/TrkB/ERK pathways, with identified Q-markers providing a foundation for optimization of quality.

**Supplementary Information:**

The online version contains supplementary material available at 10.1186/s13020-025-01219-6.

## Introduction

Sihosogansan (SHSGS), also known as Chaihu-Shugan-San, is a traditional medicine composed of seven crude herbs, namely *Bupleurum chinense* DC., *Citrus reticulata* Blanco, *Cyperus rotundus* L., *Ligusticum striatum* DC., *Citrus aurantium* L., *Paeonia lactiflora* Pall., and *Glycyrrhiza uralensis* Fisch [1]. In traditional Chinese medicine, SHSGS is used to address emotional and psychological imbalances, primarily by alleviating liver-qi stagnation [2]. Clinical studies have indicated that SHSGS has fewer side effects than selective serotonin reuptake inhibitors (SSRIs); however, current protocols require high doses (4–19.5 g/kg) and prolonged treatment periods (4–12 weeks) [3]. This extended period of therapeutic onset poses increased risks for patients with severe depression.

Recent breakthroughs in rapid-acting antidepressants, particularly ketamine and its derivatives, have highlighted the critical importance of rapid onset of depression treatment. These agents can elicit antidepressant responses within hours through novel mechanisms that increase gamma-aminobutyric acid (GABA) synaptic function and subsequently enhance synaptic plasticity [4]. Dysregulation of inhibitory GABAergic signaling, which includes reduced cortical or hippocampal GABA levels, diminished glutamate decarboxylase (GAD67), and altered GABAB receptor function, is a consistent feature of stress-related depression [5]. The GABA_B1_ receptor is a key metabotropic GABA receptor subunit that modulates synaptic transmission via Gi/o-coupled pathways, thereby influencing presynaptic release probability and postsynaptic excitability [6]. Engagement of the GABA_B1_ receptor can interface with intracellular cascades that regulate neurotrophic signaling and synaptic remodeling, including extracellular signal-regulated kinase (ERK)-dependent pathways [7]. In parallel, brain-derived neurotrophic factor (BDNF) and its high-affinity tropomyosin receptor kinase B (TrkB) are central regulators of neuroplasticity, promoting neuritogenesis, synaptogenesis, and activity-dependent structural maturation, and are repeatedly implicated in the rapid action of antidepressants [8]. Therefore, converging evidence links GABAergic tone and BDNF/TrkB/ERK signaling to mood-relevant plasticity in hippocampal circuits [4, 5].

Concerns regarding the potential for abuse and adverse effects of existing fast-acting antidepressants have intensified the search for alternative rapid-acting agents, particularly those from natural sources with established safety profiles. We hypothesized that a volatile-rich, simultaneously distilled–extracted (SDE) preparation of SHSGS delivered intranasally to favor central access could rapidly engage the GABAergic and BDNF/TrkB pathways and produce antidepressant-like effects. Traditional decoction methods predominantly yield water-soluble compounds with limited systemic bioavailability [9]. Additionally, the lack of standardization and quality control metrics has hindered the modernization of this traditional formula. The complexity of SHSGS, which contains multiple bioactive compounds, presents an opportunity for the development of rapid-acting formulations. However, the identification of quality markers (Q-markers), which serve as objective indicators of pharmaceutical quality and therapeutic efficacy, is essential to address these challenges and ensure reproducible therapeutic outcomes [10]. We further reasoned that specific monoterpenes within SHSGS would serve as bioactive Q-markers if they were consistently tracked for behavioral efficacy and mechanistic readouts.

Here, we employed a systematic approach focusing on selective extraction and optimization of intranasal delivery to enhance bioavailability in the central nervous system (CNS), along with evidence-based identification of Q-markers. Initial investigations used zebrafish and mouse models for rapid screening of anxiolytic and antidepressant effects, followed by detailed mechanistic studies examining the GABAergic and BDNF/TrkB/ERK pathways. Network pharmacology analysis integrated with traditional compatibility principles and experimental validation guided the identification of Q-markers. This investigation aimed to contribute to the growing body of evidence supporting the integration of traditional remedies with modern pharmacological insights, ultimately enhancing the control of depression and reducing suicide rates.

## Materials and methods

### Preparation of SHSGS

SHSGS was mixed from seven dried herbs (Omniherb, Daegu, Korea) including *Bupleurum chinense* DC., *Citrus reticulata* Blanco, *Ligusticum striatum* DC., *Cyperus rotundus* L., *Citrus aurantium* L., *Paeonia lactiflora* Pall., and *Glycyrrhiza uralensis* Fisch as a ratio of 3:3:2:2:2:2:1 (w/w), respectively. SHSGS (100 g) was mixed with distilled water at a ratio of 1:10 (w/v). The extraction process was performed 4 h using a simultaneous distillation–extraction (SDE) apparatus, with n-hexane as the solvent. The n-hexane was then separated using a rotary evaporator, and obtained oil was weighed (0.2 g, yield 0.62%) and stored at – 20 °C.

### Zebrafish and electroencephalogram (EEG) recording

Zebrafish (Danio rerio, AB strain) were maintained under a 14 h:10 h light: dark cycle in purified water (pH 6.5–7.5) at 27 ± 1 °C. The fish were anesthetized in 300 mL water containing 16 mg/L eugenol and treated with MK-801 (Sigma-Aldrich, St. Louis, MO, USA) to induce anxiety-like behaviors. After 30 min, SHSGS (10 mg/L) or haloperidol (9 μM, positive control) were administered. After another 30 min, the EEG was recorded for 20 min using non-invasive electrodes. An MP36 device (Biopac Systems Inc., CA, USA) was used to measure and process the EEG signals, and the fast Fourier transform method was used to analyze the frequency of the signals. The relative power spectral densities (slow oscillation, pure delta, delta, theta, alpha, beta1, beta2, beta, and slow gamma) were recorded.

### Animals and experimental design

Six-week-old male ICR mice from Koatech (Gyeonggi, Korea) were housed under a 12 h/12 h light–dark cycle (22–23 °C, 45–50% humidity) with water and standard diet (5L79, PMI Nutrition, St Louis, MO, USA) ad libitum. For the SHSGS experiment, mice (n = 7–8) were divided into five groups: CON (vehicle, intranasally), IN12.5 (12.5 mg/kg SHSGS, intranasally), IN25 (25 mg/kg SHSGS, intranasally), PO12.5 (12.5 mg/kg SHSGS, orally), and PO25 (25 mg/kg SHSGS, orally). For the combination therapy experiment, the groups (n = 7) included CON (vehicle), FLU12.5 (12.5 mg/kg fluoxetine, orally), SHSGS12.5 (12.5 mg/kg SHSGS, intranasally), and SHSGS + FLU (12.5 mg/kg SHSGS intranasally plus 12.5 mg/kg FLU via orally). For the Q-marker compound experiment, the groups (n = 5) included CON (vehicle, intranasally), MEM (3.125 mg/kg memantine, intraperitoneal), and compound groups (3.125 or 6.25 mg/kg, intranasally) were administered 30 min before being given the tail suspension test (TST) or forced swimming test (FST) (Fig. 5A). Besides, to confirm the underlying mechanisms of SHSGS and its compounds, the GABA_B1_ receptor antagonist CGP 35348 (Abcam, Cambridge, UK) was used. ICR mice were arranged into the following groups (n = 5): CON (vehicle, intranasally), SHSGS (12.5 mg/kg, intranasally), and compound-treated groups (6.25 mg/kg, intranasally) with or without CGP 35348 (100 mg/kg, intraperitoneally). CGP 35348 (dissolved in saline) was administered 30 min before drug administration, and the FST was conducted 30 min after drug administration (Fig. 5B). Three compounds—β-pinene (CFN93287), terpinen-4-ol (CFN94877), and α-terpineol (CFN80110) (≥ 98% purity) were purchased from ChemFaces (Wuhan, Hubei, China). SHSGS and its three compounds were dissolved in 3% Tween 80 in saline solution as the vehicle. Fluoxetine (FLU) and memantine (MEM) were purchased from Sigma-Aldrich (St. Louis, MO, USA), and dissolved in saline. CGP 35348 (≥ 98% purity) was purchased from TargetMol (123,690–79-9, Boston, MA, USA), and dissolved in saline. All experiments were conducted following the guidelines of the Institutional Animal Care and Use Committee of Dongguk University (IACUC-2023–18).

### Open field test (OFT)

Mice were Habituated to the test room for 2 h prior to testing. The mice were then placed in the center of an OFT box (30 cm × 30 cm × 30 cm) for 10-min exploration. The box was cleaned with 70% ethanol between trials. Smart V3.0 software (Panlab, Barcelona, Spain) recorded entries, time, and distances in the center zone (18 cm × 18 cm square in the center of the arena).

### Tail suspension test (TST)

Mice were suspended by adhesive tape (15 cm length), with their tails attached to a bar 50 cm above the ground. Using the SMART V3.0 tracking system, immobility time (no forelimb movement) was recorded for 6 min.

### Enzyme-linked immunosorbent assay (ELISA)

The levels of GABA and BDNF in the hippocampus (HPC) were assessed using ELISA kits following the guidelines provided by the manufacturer. The ELISA kits including GABA (EU2602) and BDNF (EM0020) were obtained from FineTest (Wuhan Fine Biotech, China). Absorbance at 450 nm was measured using a Tecan microplate reader (Männedorf, Switzerland).

### Hematoxylin and eosin (H&E) staining and lactate dehydrogenase (LDH) assay

After 24 h administration of SHSGS (25 mg/kg, intranasally) and its compounds (6.25 mg/kg, intranasally), H&E staining and LDH assay were conducted to assess the toxicity in the nasal mucosa and olfactory bulbs, respectively. For H&E staining, nasal mucosal tissues were fixed in 4% paraformaldehyde, embedded in paraffin, and sectioned at 5 μm thickness. Sections were stained with hematoxylin and eosin, and histological images were captured using BioTek Lionheart FX automated microscope with Gen5 software (Ver. 3.08). For the LDH assay, olfactory bulbs were isolated and homogenized in ice-cold lysis buffer. The homogenates were centrifuged at 12,000 ×*g* for 10 min at 4 °C, and the supernatants were collected for analysis. LDH activity was measured using a commercially available LDH cytotoxicity assay kit (ab102526, Abcam, Cambridge, UK) according to the manufacturer’s instructions. Absorbance was read at 450 nm using a Tecan microplate reader (Männedorf, Switzerland).

### Immunofluorescence analysis

Mouse brains were post-fixed for 24 h and embedded in paraffin. Brain Sects. (10 μm) were prepared using a cryo-ultramicrotome (Leica, Wetzlar, Germany), and hippocampal regions (CA1 and dentate gyrus) were selected. After deparaffinization and H_2_O_2_ treatment, sections were incubated with the primary antibodies, mouse anti-NeuN (MAB377, Millipore, Bedford, MA, USA) and rabbit anti-BDNF (PA5-85,730, Thermo Fisher Scientific, Waltham, MA, USA) overnight at 4 °C. The secondary antibodies were FITC-conjugated goat anti-rabbit IgG (A-11011, Alexa Fluor^®^ 568, Invitrogen, Carlsbad, CA, USA) and goat anti-mouse IgG (A-11001, Alexa Fluor^®^ 488, Invitrogen, Carlsbad, CA, USA) at 37 °C for 1 h. Sections were mounted in DAPI-containing medium (Vector Laboratories, CA, USA) and imaged using a Nikon fluorescence microscope with NIS-Elements BR 4.50 software (Nikon, Tokyo, Japan) at 400 × magnification.

### Western blotting

Lysates (15–25 μg/lane) were separated on SDS-PAGE with 5% stacking gel and 10–15% resolving gel, based on targets. The proteins were transferred onto PVDF membranes (Merck Millipore, Carrigtwohill, Ireland) and blocked with 5% skim milk for 2 h. The membranes were then incubated with primary antibodies (overnight, 4 °C) and secondary antibodies (1 h, RT). The antibodies were: rabbit anti-GABABR1 (A10504, Abclonal, Woburn, MA, USA), rabbit anti-GAD67 (GTX101881, GeneTex, Irvine, CA, USA), rabbit anti-BDNF (ab108319, Abcam, Cambridge, UK), rabbit anti-p-ERK (9101, Cell Signaling Technology, Danvers, MA, USA), mouse anti-β-actin (A1978, Sigma-Aldrich, St. Louis, MO, USA), anti-rabbit IgG, HRP-linked whole antibody (NA934, Amersham ECL, Little Chalfont, UK), and goat anti-mouse IgG (BML-SA204-0100, Enzo Life Sciences, Farmingdale, NY, USA).

### Gas chromatography–mass spectrometry (GC–MS) analysis

The components of SHSGS were analyzed using GC–MS (8890GC/5977MSD, Agilent, Santa Clara, CA, USA) with the instrument parameters described in Supplementary Table S1 and identified using the National Institute of Standards and Technology Mass Spectra Library.

### Prediction of SHSGS’s potential antidepressant targets and Q-markers

#### Compound and target identification

Following GC/MS analysis, SHSGS compounds were detected, and their targets were retrieved from the Traditional Chinese Medicine Systems Pharmacology Database and Analysis Platform (TCMSP, https://tcmsp-e.com/tcmsp.php). Separately, depression targets were obtained from the DisGeNET database (ID: C0011570, https://disgenet.com/) and Therapeutic Target Database (ID: ICD-9:311, https://idrblab.net/ttd/). The key step involved identifying the overlapping targets between the SHSGS compounds and depression targets, followed by the retrieval of specific compounds associated with these common targets.

#### Enrichment analysis

With the identified overlapping targets between SHSGS and depression, Kyoto Encyclopedia of Genes and Genomes (KEGG) enrichment and Gene Ontology (GO) analyses were performed using SRplot tool (https://www.bioinformatics.com.cn/srplot). The most involved KEGG pathways and top 10 GO terms (including biological processes, cellular components, and molecular functions) were automatically selected by the tool, being used as references for the in vitro and in vivo mechanistic studies.

#### Network construction

To create the compound-target-pathway network, two Excel files were prepared: one for compound-target interactions and another for target-pathway interactions. Each file was organized into columns specifying the source node (compounds or pathways) and the target node (proteins), which were then imported into the Cytoscape software (v.3.8.2). Through the topological analysis function in Cytoscape, the degree centrality (DC) and closeness centrality (CC) of each compound were analyzed. Compounds with a DC value greater than 10 and a CC value greater than 0.4 were considered significant and were used as one of the criteria for selecting the candidate Q-markers of SHSGS.

### Molecular docking analysis

The 3D structure of GABA type B receptor subunit 1 (GABABR1) (ID: 4MS4) was obtained from RCSB Protein Data Bank, while those of β-pinene (ID: 14,896), terpinen-4-ol (ID: 11,230), α-terpineol (ID: 17,100), and baclofen (ID: 2284) were from PubChem. Proteins were processed using LigPrep module of the Schrodinger suite 2021–3 by removing ions, and water molecules. Additionally, the refinement processes, including the suitable chiral position, ionization and protonation state, continued by employing Epik at a target pH of 7.0 ± 2.0 with OPLS3 force field. The Autogrid option allows for the selection of the binding site, and the configuration of the grid size was set to 20 × 20 × 20 points around the reference ligand. The glide extra-precision (XP) docking was performed, and all other parameters were default settings. 2D images were generated using Discovery Studio Visualizer 2021. After that, all docked complexes were then subjected to MM-GBSA calculation by using a prime module from the Schrödinger suite with the force field OPLS3 [11].

### Molecular dynamics (MD) simulation

MD simulation was performed with Desmond through the Schrödinger Maestro interface academic software version (2020–1) accessible online (https://www.schrodinger.com/). A water box (cubic) of 20 Å as boundary conditions was set utilizing the pre-defined TIP3P water model employing force field OPLS3 [12]. The docked complex's overall charge was balanced by adding 0.15 M Na + and Cl − ions to the solution. The NPT setup was applied with the following parameters held constant: temperature 300 K, pressure 1.01325 bar. The system underwent two stages of energy minimization (restrained and unrestrained) using an energy gradient convergence threshold of 1 kcal/mol/Å. After that, four stages of short MD runs (12 ps, 12 ps, 12 ps, and 24 ps) were proceeded with progressively reduced restraints and elevated temperature each using the NPT ensemble at 10, 10, 300, and 300 K, respectively, with the default relaxation strategy in Desmond. Finally, MD simulation was run for 200 ns at 300 K and 1 bar of pressure, and each trajectory was recorded at intervals of 25 ps for the final study.

### Cell culture and treatment conditions

PC12 and BV2 cells were cultured in RPMI and DMEM media respectively, supplemented with 10% FBS and 1% penicillin/streptomycin (Welgene, Gyeongsangbuk, Korea) at 37 °C. For viability assays, cells were treated with dimethyl sulfoxide, SHSGS (0.1–100 μg/mL), FLU (1.25 μM), or compounds (6.25–50 μg/mL) for 24 h. Viability was measured using WST substrate (EZ-Cytox kit, DoGenBio, Seoul) with a Tecan microplate reader (Männedorf, Switzerland). The absorbances were measured at 450 nm (detection wavelength) and 650 nm (reference wavelength).

For neuroprotection studies, PC12 cells were pre-treated with SHSGS or compounds for 3 h prior to 24 h corticosterone (100 μM) stimulation, and cell viability was measured using the EZ-Cytox kit (DoGenBio, (Seoul, Korea). For anti-inflammatory studies, BV2 cells were pre-treated with SHSGS and compounds 3 h prior to lipopolysaccharide (1 μg/mL) stimulation for 24 h. Supernatants were collected for ELISA after centrifugation (1,500 rpm, 4 °C, 10 min). Mouse interleukin 6 (IL-6) and tumor necrosis factor alpha (TNF-α) ELISA kits were from LABISKOMA (Seoul, Korea). The absorbance was measured at 450 nm using a Tecan microplate reader.

### Cell apoptosis analysis

For cell apoptosis analysis, only the neuroprotective doses of SHSGS and its compounds from WST assay were tested. PC12 cells were pre-treated with SHSGS or its compounds for 3 h, followed by CORT (100 μM) for 24 h. Apoptosis was assessed using the Muse Annexin V & Dead Cell Assay (Millipore, Billerica, MA, USA) according to the manufacturer’s protocol. Briefly, cells were washed with 1X PBS and resuspended in RPMI containing 1% FBS. After centrifugation, the cell suspensions were adjusted to a final volume of 100 µL (with 60.000 cells) and incubated with 100 µL of Muse Annexin V and Dead Cell reagent. Samples were mixed thoroughly, incubated for 20 min at RT in the dark, and analyzed for apoptotic cell concentration using the Muse Cell Analyzer (Millipore, Billerica, MA, USA).

### Blood–brain barrier (BBB) permeability assay

The BBB permeability of SHSGS compounds was assessed using the PAMPA-BBB Kit (PMBBB-096, BioAssay Systems, Hayward, CA, USA) according to the manufacturer’s instructions. Briefly, test compounds and reference standards (promazine hydrochloride and diclofenac sodium) were diluted to 500 µM in PBS. Donor wells coated with a Lipid membrane were loaded with compound solutions, then assembled with acceptor wells containing PBS. After 18 h of incubation at RT, 100 µL from the acceptor wells was transferred to a UV-transparent plate, and absorbance was measured at the compounds'peak absorbance ranging, from 200 to 500 nm. Apparent permeability coefficients were calculated as directed.

### Drug affinity responsive target stability (DARTS)

DARTS experiments were performed as previously described with minor modifications [13]. Briefly, SH-SY5Y cell lysates were incubated with either DMSO or test compounds (6.25 and 12.5 μg/mL) for 1 h at RT, followed by proteolysis with pronase (10,165,921,001, Roche, Mannheim, Germany) at concentrations of 4 and 8 μg/mL in TNC buffer for 30 min at RT. Reactions were stopped by adding loading buffer and heating the samples at 100 °C for 15 min. Protein expression was analyzed by western blotting using the following primary antibodies: rabbit anti-GABABR1 (A10504, Abclonal, Woburn, MA, USA), and mouse anti-β-actin (A1978, Sigma-Aldrich, St. Louis, MO, USA). HRP-conjugated secondary antibodies included anti-rabbit IgG, HRP-linked whole antibody (NA934, Amersham ECL, Little Chalfont, UK), and goat anti-mouse IgG (BML-SA204-0100, Enzo Life Sciences, Farmingdale, NY, USA).

### Primary hippocampal neuronal cell culture

E-17 pregnant Sprague–Dawley mice were maintained under a 12 h light/dark cycle with food and water at room temperature (RT). Primary hippocampal neurons were collected from rat embryos as previously described [14]. Pregnant rats were anesthetized with isoflurane and euthanized. Embryo hippocampi were isolated in cold Hank’s solution after removal of the meninges. Neurons were isolated using trypsin EDTA (0.25%) for 12 min at 37 °C and triturated using fire-polished Pasteur pipettes. Cells were seeded on poly-D-lysine-coated coverslips at 1.0 × 101 cells/cm2 for morphometry and 2.0 × 104 cells/cm2 for synaptogenesis. Plating media was treated with SHSGS (1- 40 μg/mL), β-pinene, terpinen-4-ol, α-terpineol (1–50 μg/mL), scoparone (SCOP) (37.5 μM), and FLU (1.25 μM) before seeding. Fresh medium with treatments was supplied every four days. Experiments were conducted in accordance with the Institutional Animal Care and Use Committee of Dongguk University (IACUC-2023–18).

### Immunocytochemistry

Hippocampal neurons were fixed with 4% paraformaldehyde on the 3rd day in vitro (DIV3) and DIV14. After 1 h blocking with 5% goat serum, neurons were stained with primary antibodies: rabbit anti-BDNF (PA5-85,730, Invitrogen, CA, USA), mouse anti-tubulin α-subunit (4A1, Developmental Studies Hybridoma Bank, IA, USA), rabbit anti-p-Erk1/2 (9101, Cell Signaling, MA, USA), mouse anti-TrkB (sc-377218, Santa Cruz, CA, USA), rabbit anti-actin (A2066, Sigma, MO, USA), rabbit anti-GABABR1 (A10504, Abclonal, Woburn, MA, USA), mouse anti-glutamic acid decarboxylase (GAD-6, Developmental Studies Hybridoma Bank, IA, USA). The secondary antibodies were Alexa Fluor 488/568 goat anti-mouse/rabbit IgG (A-11001/A-11011, Invitrogen, CA, USA). Images were captured using a Leica DFC3000G fluorescence microscope equipped with a Sony CCD sensor and LasX software (3.7.2.22383). Images of synaptic puncta were acquired using an Olympus microscope (Tokyo, Japan) equipped with a DP74 CMOS camera and CellSens software (1.18). Morphometric analysis and counting of synaptic puncta were performed using ImageJ software (version 1.49).

### Intracellular calcium assays

Primary hippocampal neuronal cells (on DIV7) were treated with the GABA_B1_ receptor antagonist CGP 35348 at 200 μM and TrkB inhibitor ANA-12 (Tocris Biosciences, Fischer Scientific, USA) at 1 μM. After 48 h of incubation, cells were rinsed with 1 × DPBS and treated with 1 μM Fluo-4 (MedChemExpress, HY-101896) for 40 min at 37 °C. Subsequently, the cells were rinsed twice with 1 × DPBS and observed under a fluorescence microscope with illumination at 488 nm (green fluorescence). The mean intensity was then measured using ImageJ software to quantify intracellular calcium levels.

### Statistical analysis

GraphPad Prism 8.0.1 (GraphPad Software, San Diego, CA, USA) was used for statistical analysis. Data normality was assessed using the Shapiro–Wilk test and variance homogeneity using the Brown-Forsythe test. For a normal distribution with homogeneous variance, one-way analysis of variance (ANOVA) with Dunnett's post hoc test was performed. For a normal distribution with heterogeneous variance, Welch's ANOVA with Dunnett T3 post hoc test was used. Non-normal distributions were analyzed using the Kruskal–Wallis test with Dunn's post hoc test. All data are presented as mean ± standard error of the mean (SEM). Statistical significance was set at p ≤ 0.05.

## Results

### SHSGS rapidly ameliorates anxiety-related EEG patterns in MK-801-treated zebrafish

**Fig. 1:**
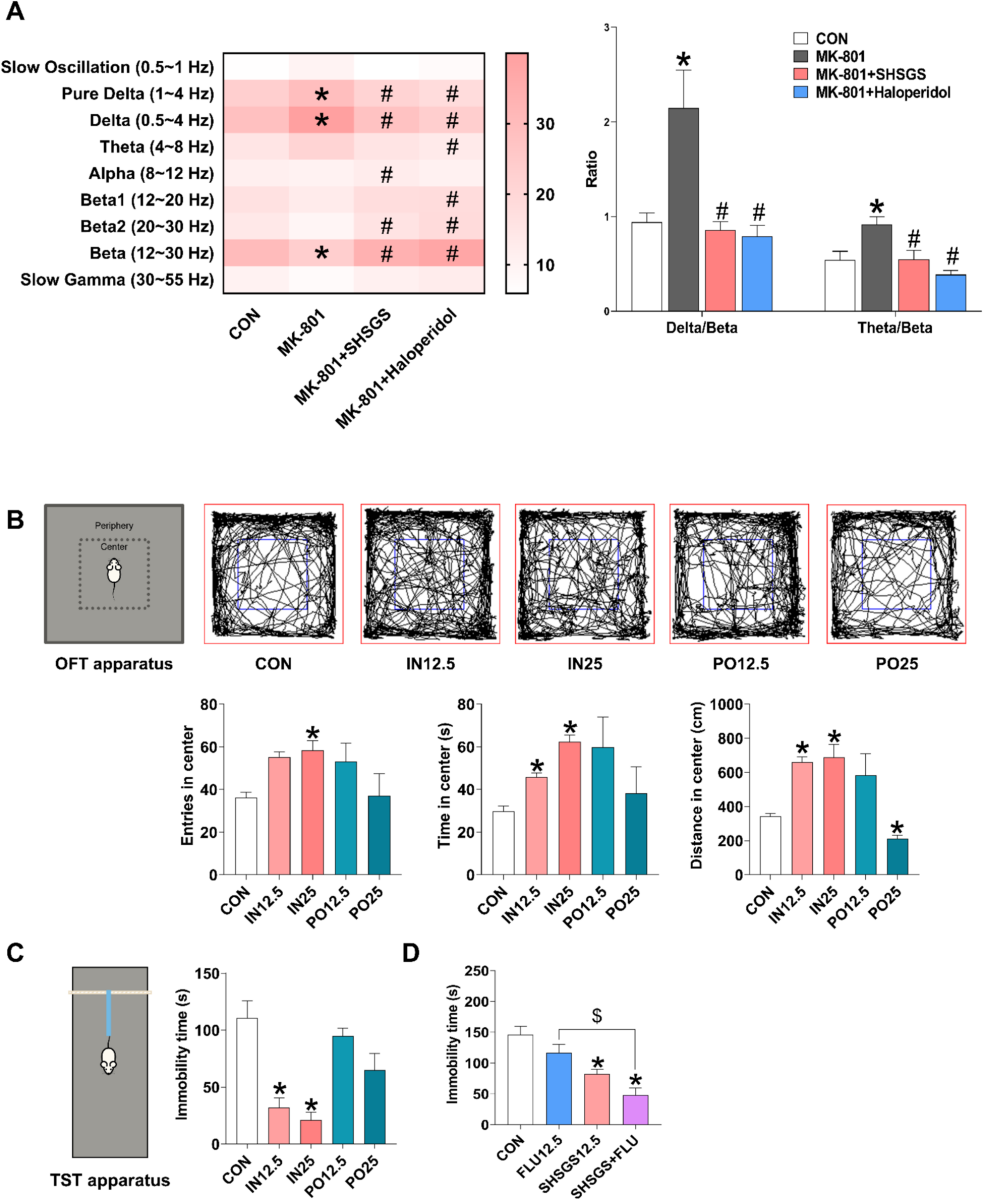
Rapid anti-anxiety and anti-depressive effects of SHSGS in vivo. **A** Effects of SHSGS (10 mg/L) on EEG signals in an MK-801-induced anxiety-like model in zebrafish shown by a heat map representing the EEG relative power spectrum (Left) and Delta/beta and theta/beta ratios (Right). Data are presented as mean ± SEM (n = 8 per group). Effects of SHSGS on mouse behaviors in **B** the OFT and **C** TST. **D** Effects of SHSGS, FLU, and their combined treatment in the TST. Data are presented as mean ± SEM (n = 6–8 per group). *p < 0.05 vs. CON, ^#^p < 0.05 vs. MK-801, ^$^p < 0.05 vs. FLU

To examine the rapid anxiolytic effect of SHSGS, zebrafish were pretreated with MK-801 to induce anxiety-like behaviors, followed by treatment with 10 mg/L SHSGS. The EEG measurement after 30 min showed that MK-801 significantly increased the relative power spectral densities of delta and pure delta, while obviously decreased that of beta; however, all these changes were remarkably reversed in groups treated with SHSGS and the positive control haloperidol. Moreover, significant elevations in the densities of alpha and beta2 were found in the SHSGS treatment group compared to those in the model group (Fig. 1A). The ratios of some brainwave frequencies were calculated from the relative power spectral densities. SHSGS and haloperidol significantly reduced MK-801-induced elevations in the delta/beta and theta/beta ratios in zebrafish (Fig. 1A).

### Intranasal SHSGS exhibits rapid anxiolytic and antidepressant effects

To further investigate the rapid effect of SHSGS, the behavior of mice after a single treatment was assessed using the OFT. Mice administered SHSGS intranasally, but not orally, had significantly higher number of entries, time, and distance traveled in the central zone of the arena compared to the CON group (Fig. 1B). These results indicate that at the same time point, duration, and dosage of treatment, the intranasal route exerted a more rapid and faster anxiolytic-like effect in mice compared to oral administration.

To assess the potential rapid antidepressant effects of SHSGS, the TST was conducted under the same treatment conditions as in the OFT. Although both doses of intranasal SHSGS (12.5 and 25 mg/kg) significantly reduced the immobility time of mice under suspension, no effects of oral SHSGS were observed (Fig. 1C). These results indicate that intranasal SHSGS can reduce despair-related behavior in mice and further affirms its rapid action compared with oral administration. To explore the therapeutic advantages of SHSGS, we evaluated whether its intranasal administration could enhance the conventional SSRI treatment. In the TST, while FLU treatment showed no significant effect at 30 min post-administration, combined administration of intranasal SHSGS and oral FLU demonstrated significantly decreased immobility time compared to either CON or FLU alone, or greater effect size than SHSGS alone, suggesting a synergistic effect of the two treatments (Fig. 1D).

### GC–MS analysis reveals predominant monoterpenes in SHSGS composition

**Fig. 2:**
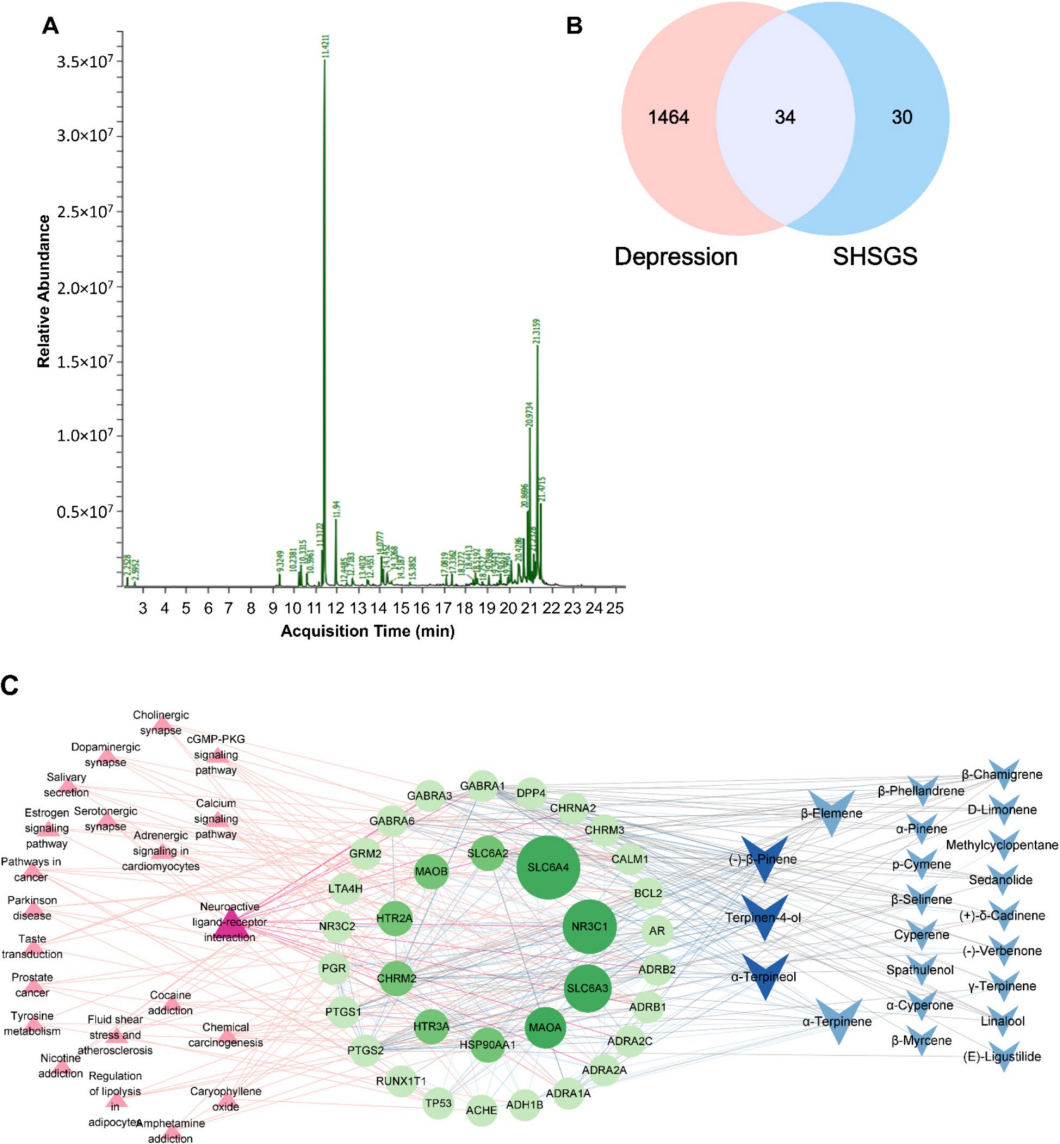

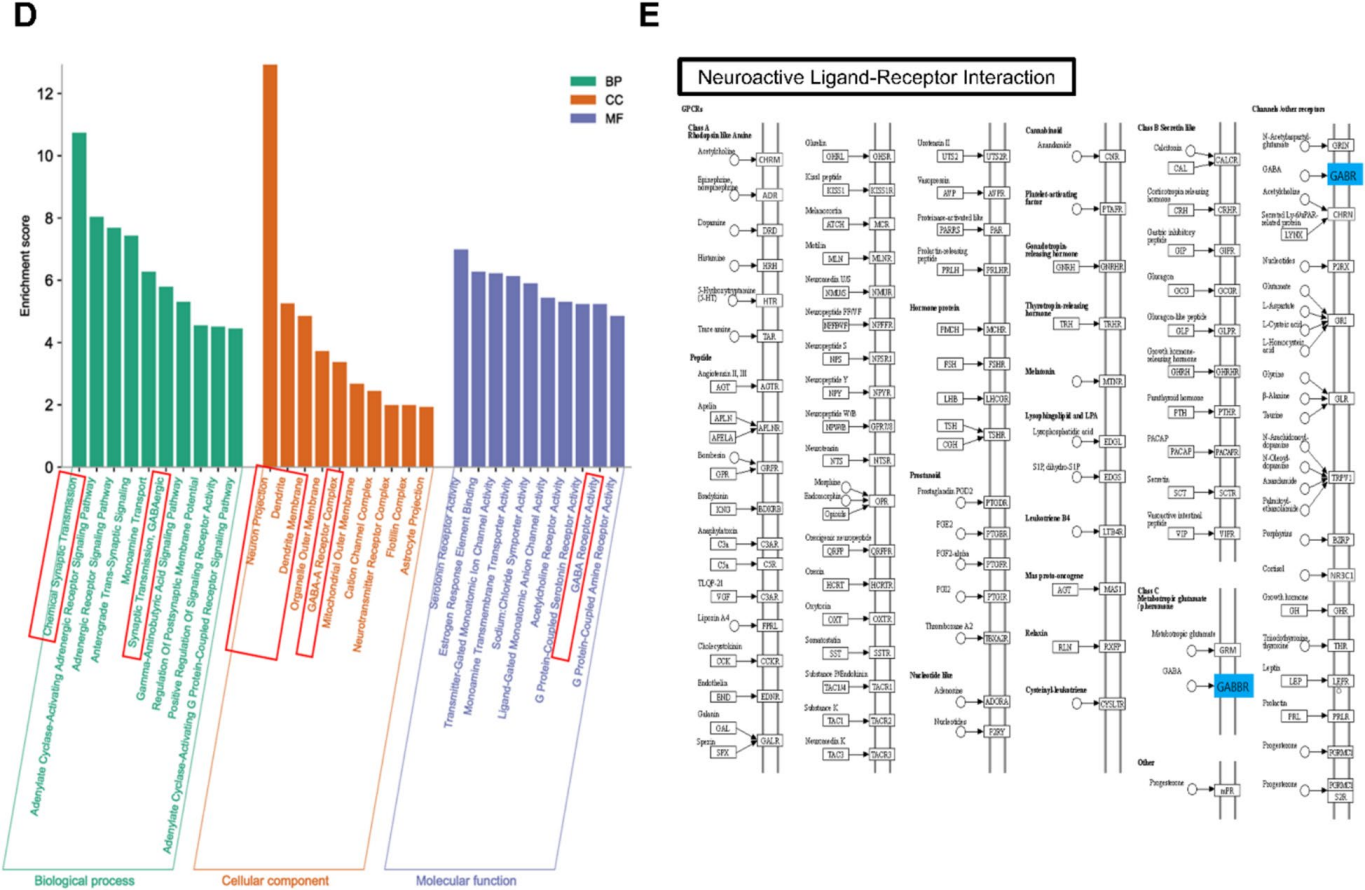
In silico study with SHSGS components. **A** GC–MS chromatogram of SHSGS essential oil. **B** A Venn diagram shows the intersection between SHSGS targets and depression-related targets. **C** Compound-target-pathway network of the common targets between SHSGS and depression. **D** GO enrichment analysis. **E** Related targets involved in the neuroactive ligand-receptor interaction pathway from KEGG

GC/MS was used to analyze the composition of SHSGS. A total of 35 compounds were detected in the extracted composition, belonging to various groups, such as alkanes (methylcyclopentane, cyclohexane), monoterpenes (α-pinene, p-cymene), or phthalides (3-n-butylphthalide, ligustilide), with compounds from the monoterpenes group being the predominant (Fig. 2A). The top 10 major compounds in SHSGS are D-limonene (38.4%), (E)-ligustilide (13.4%), cyperotundone (6.6%), α-cyperone (4%), 6-butyl-1,4-cycloheptadiene (3.9%), ɣ-terpinene (3.1%), p-cymene (1.6%), terpinen-4-ol (1.5%), sedanolide (1.4%), and 3-n-butylphthalide (1.3%) (Table 1).

**Table 1:** Volatile constituents of SHSGS with the corresponding retention time and concentration

No	Compound	Retention time (min)	Relative content (%)
1	D-Limonene	11,4211	38,4
2	(E)-Ligustilide	21,3159	13,4
3	Cyperotundone	20,9734	6,6
4	α-Cyperone	21,4715	4
5	6-Butyl-1,4-cycloheptadiene	20,8696	3,9
6	γ-Terpinene	11,94	3,1
7	p-Cymene	11,3122	1,6
8	Terpinen-4-ol	14,0777	1,5
9	Sedanolide	21,2328	1,4
10	3-n-Butylphthalide	20,4286	1,3
11	α-Terpineol	14,3268	1,2
12	(−)-β-Pinene	10,3315	1,1
13	β-Myrcene	10,5961	0,8
14	Myrtanal	14,1452	0,8
15	α-Pinene	9,3249	0,7
16	β-Phellandrene	10,2381	0,7
17	Linalool	12,7183	0,7
18	Caryophyllene oxide	19,614	0,6
19	Cyperene	17,3362	0,5
20	β-Selinene	18,4413	0,5
21	(−)-trans-Pinocarveol	13,4551	0,4
22	β-Chamigrene	18,5192	0,4
23	Cyperene epoxide	19,0588	0,4
24	Humulene epoxide II	19,9461	0,4
25	Methylcyclopentane	2,2528	0,3
26	(−)-Verbenone	14,5187	0,3
27	β-Elemene	17,0819	0,3
28	α-Terpinene	11,1513	0,2
29	Isoterpinolene	12,4485	0,2
30	(1R)-(+)-Nopinone	13,4032	0,2
31	(−)-cis-Myrtanol	15,3852	0,2
32	cis-β-Copaene	18,3272	0,2
33	Spathulenol	19,5413	0,2
34	Cyclohexane	2,5952	0,1
35	(+)-δ-Cadinene	18,7527	0,1

### Network analysis reveals GABAergic and neuroplasticity pathways as key SHSGS targets

To construct the network of SHSGS compounds and potential targets, the target genes of the 35 compounds from the GC/MS data in the TCMSP database were identified (Supplementary Table S2). Only 23 compounds had retrievable targets in the database, with a total of 64 targets. There were 1,498 targets related to"Depression"from the DisGeNET and Therapeutic Target databases. Among them, 34 overlapping targets were identified, and 22 corresponding compounds from 34 targets were retrieved (Fig. 2B). Using these common targets, KEGG enrichment analysis was performed to predict the signal pathways related to the antidepression-like effect of SHSGS and the top five signal pathways were “neuroactive ligand-receptor interaction”, “serotonergic synapse”, “chemical carcinogenesis”, “calcium signaling pathway”, and “taste transduction” (Supplementary Table S3). As shown in Fig. 2C, a compound-target-pathway network was constructed, containing 22 compounds (blue nodes), 34 proteins (green nodes), and 21 pathways (pink nodes) connected by 365 edges. Larger or darker nodes indicate higher node degree values or more significant roles in the network.

Thirty items related to biological processes, cellular components, and molecular functions were screened in the GO analysis (Supplementary Table S4). Among them, pathways that appeared with higher frequency or scores, such as “chemical synaptic transmission”, “synaptic transmission, GABAergic”, “neuron projection”, “dendrite”, and “dendrite membrane”, “GABA-A receptor complex”, and “GABA receptor activity” were identified (Fig. 2D). When further analyzing the top pathway'neuroactive ligand-receptor interaction'suggested by KEGG, GABA receptors were also found to be involved (Fig. 2E). These results suggest that the pathways involved in SHSGS are related to the GABAergic system and neuroplasticity.

### SHSGS modulates GABAergic and BDNF/TrkB signaling in the hippocampus (HPC)

Our prior computational predictions indicate that the pathways involved in SHSGS effects are GABA receptors, synaptic transmission, and neuroplasticity. To select more specific biomarkers associated with these mechanisms, a comprehensive literature review was performed, with a particular focus on the reported mechanisms of the rapid-acting antidepressant ketamine [4, 5, 15, 16]. Accordingly, we examined the expression of GABABR1, GAD67, BDNF, and phospho-extracellular signal-regulated protein kinase (p-ERK) to investigate the mechanisms underlying the fast-onset effects of SHSGS.

**Fig. 3:**
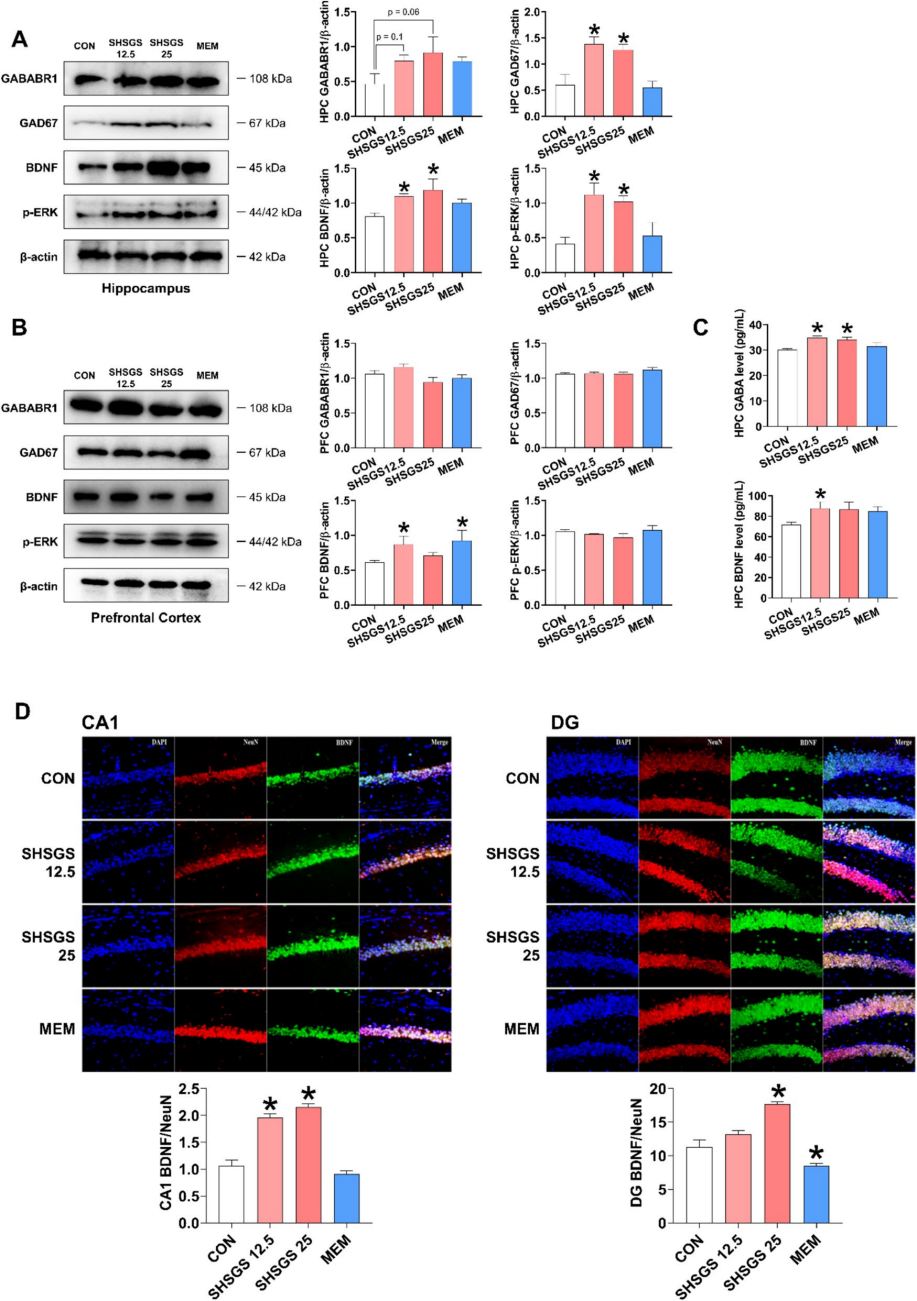
Effects of SHSGS on protein expression in the brain of mice. Protein expression of GABABR1, GAD67, BDNF, and p-ERK in the **A** HPC and **B** PFC. **C** GABA and BDNF levels in the HPC. Data are presented as mean ± SEM (n = 5 per group). **D** Immunofluorescence analysis of BDNF expression in the hippocampal CA1 and DG. Data are presented as mean ± SEM (n = 3 per group). *p < 0.05 vs CON

In the HPC, SHSGS at 12.5 and 25 mg/kg significantly increased the expression of all markers, except GABABR1 (Fig. 3A). In contrast, the treatments did not alter the expression of these markers in the prefrontal cortex (PFC). However, SHSGS (12.5 mg/kg) and MEM significantly increased BDNF expression (Fig. 3B). Subsequently, ELISA assays were conducted to assess the regulation of SHSGS on hippocampal neurotransmitters. SHSGS at 12.5 mg/kg also remarkably increased the protein levels of hippocampal GABA and BDNF (Fig. 3C). Immunofluorescence labelling was performed to examine the expression of BDNF in the hippocampal CA1 and dentate gyrus (DG). NeuN, as a specific neuronal marker, was used to identify neurons. The results demonstrated that across both the hippocampal regions examined, BDNF expressions were significantly elevated in the SHSGS12.5 or SHSGS25 groups compared to those in the control group (Fig. 3D).

### Terpinen-4-ol, α-terpineol, and (−)-β-pinene are identified as bioactive Q-markers of SHSGS

By inputting compound-target interaction data into Cytoscape, the DC and CC of each compound were analyzed. DC is a measure of the number of direct connections (or edges) a node has in a network, whereas CC reflects the mean shortest path length from a given node to all other nodes in the network. The higher the values of these two metrics for a compound, the greater its contribution to the therapeutic effect of the total extract on the target diseases [17]. As in Table 2, α-terpinene, terpinen-4-ol, α-terpineol, (-)-β-pinene, β-elemene, and β-chamigrene are compounds with degree values greater than 10, as well as having higher CC values than the other components in the network, suggesting a more important role in the network. When compared with the GC/MS results, terpinen-4-ol, α-terpineol, and (−)-β-pinene Had relative contents greater than 1%, whereas β-chamigrene, β-elemene, and α-terpinene were 0.4%, 0.3%, and 0.2%, respectively (Table 1). Thus, only terpinen-4-ol, α-terpineol, and (−)-β-pinene were selected as the three candidate Q-markers of SHSGS for further consideration.

**Table 2:** Topological analysis of SHSGS compounds in the compound-target network

Compound	Degree Centrality	Closeness Centrality
α-Terpinene	18	0.5
Terpinen-4-ol	15	0.474576271
α-Terpineol	14	0.466666667
(−)-β-Pinene	13	0.459016393
β-Elemene	12	0.451612903
β-Chamigrene	11	0.444444444
β-Selinene	9	0.4375
Caryophyllene oxide	7	0.411764706
D-Limonene	6	0.411764706
Sedanolide	6	0.411764706
(−)-Verbenone	5	0.4
(+)-δ-Cadinene	5	0.405797101
Linalool	5	0.4
γ-Terpinene	5	0.405797101
α-Pinene	4	0.394366197
β-Phellandrene	4	0.394366197
Cyperene	3	0.388888889
Spathulenol	3	0.388888889
(E)-Ligustilide	2	0.383561644
α-Cyperone	2	0.383561644
Methylcyclopentane	1	0.245614035
p-Cymene	1	0.329411765
β-Myrcene	1	0.378378378

#### SHSGS candidate Q-markers exert neuroprotective and anti-neuroinflammatory effects, and exhibit BBB permeability in vitro

**Fig. 4:**
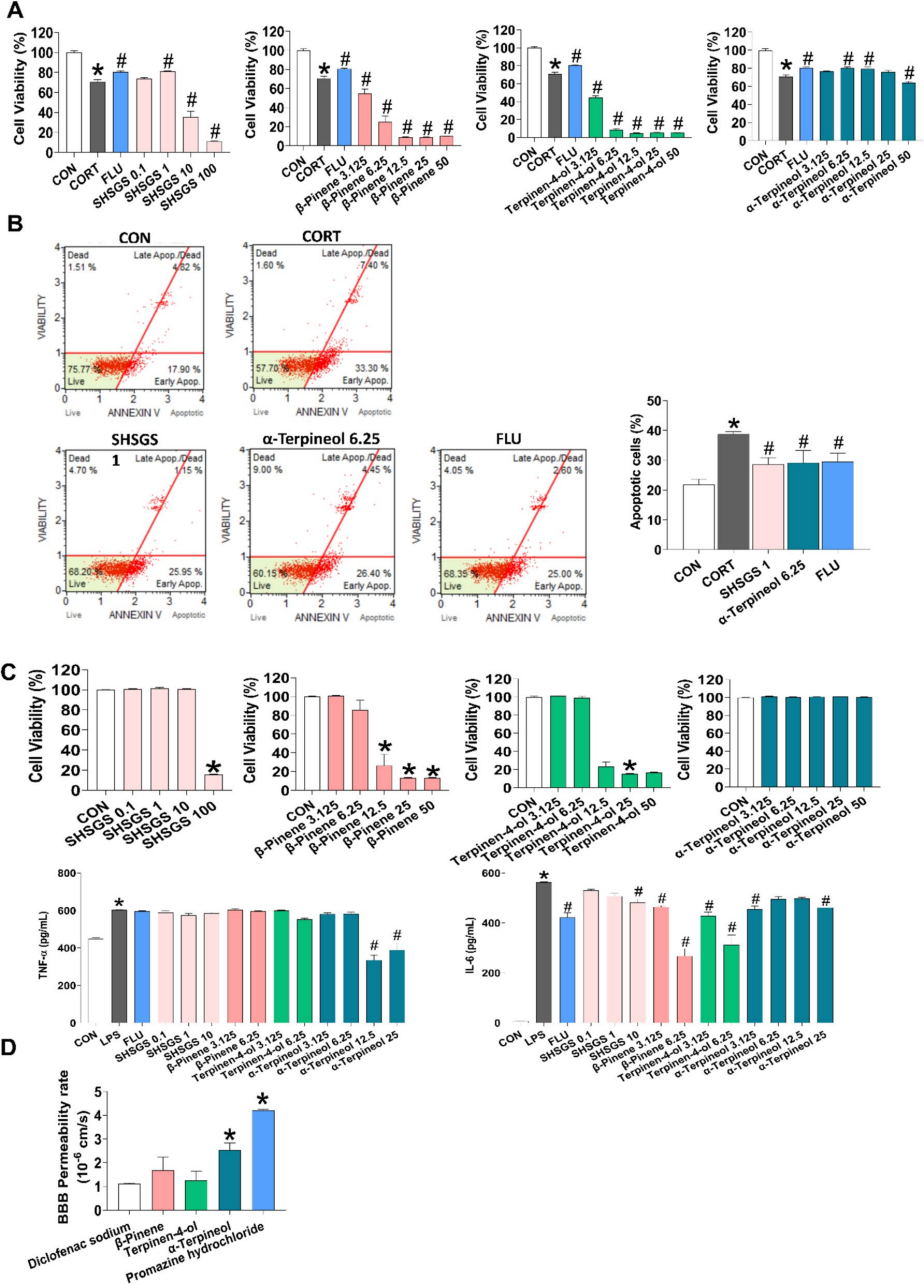
In vitro effects of SHSGS compounds. Effects of SHSGS compounds on CORT (100 µM)-induced cytotoxicity in PC12 cells via **A** WST and **B** MUSE analysis, and **C** LPS (1 µg/mL)-induced neuroinflammation in BV2 cells. FLU (1.25 µM) was the positive control. **D** In vitro BBB permeability rate of SHSGS compounds. Data are presented as mean ± SEM (n = 3 per group). * *p* < 0.05 vs. CON, # *p* < 0.05 vs. CORT/LPS-treated cells

First, the activity of terpinen-4-ol, α-terpineol, and (-)-β-pinene were tested on an LPS-induced neuroinflammatory model in BV2 cells and CORT-induced neurotoxicity model in PC12 cells, as these symptoms have been shown to be related to the pathogenesis of depression [18, 19]. Additionally, the total extract of SHSGS in the experiment was included to compare its effectiveness with that of the individual compounds. Incubation with 100 µM CORT reduced PC12 cell viability to approximately 70% compared to the CON group, whereas pre-treatment with SHSGS at 1 µg/mL prevented this effect. Among the three compounds, only α-terpineol at 6.25 and 12.5 µg/mL reversed the effect of CORT stimulation. Terpinen-4-ol and (-)-β-pinene were highly toxic within the dose range of 3.125—50 µg/mL (Fig. 4A). Consistently, 100 µM CORT induced apoptosis in PC12 cells, which was also improved by 1 µg/mL SHSGS and 6.25 µg/mL α-terpineol (Fig. 4B). Under LPS treatment (1 µg/mL), BV2 cells had substantial elevated production of TNF-α and IL-6. While SHSGS at 10 µg/mL slightly reduced only the IL-6 level, α-terpineol (25 µg/mL) can reverse the releases of both pro-inflammatory cytokines. In comparison, terpinen-4-ol and (-)-β-pinene at 6.25 µg/mL exerted greater effects in reducing IL-6, but not TNF-α (Fig. 4C).

Figure 4D shows BBB permeability rates of selected SHSGS compounds and reference drugs as determined by the PAMPA-BBB assay. Diclofenac sodium, used as a low-permeability control, exhibited the lowest permeability rate (1.12 × 10⁻⁶ cm/s). SHSGS compounds showed moderate permeability, with rates ranging between 1.27 and 2.55 × 10⁻⁶ cm/s. Among that, only α-terpineol exhibited significantly higher BBB permeability than the low-permeability control.

#### SHSGS candidate Q-markers produce antidepressant-like effects and show no acute toxicity in vivo

**Fig. 5:**
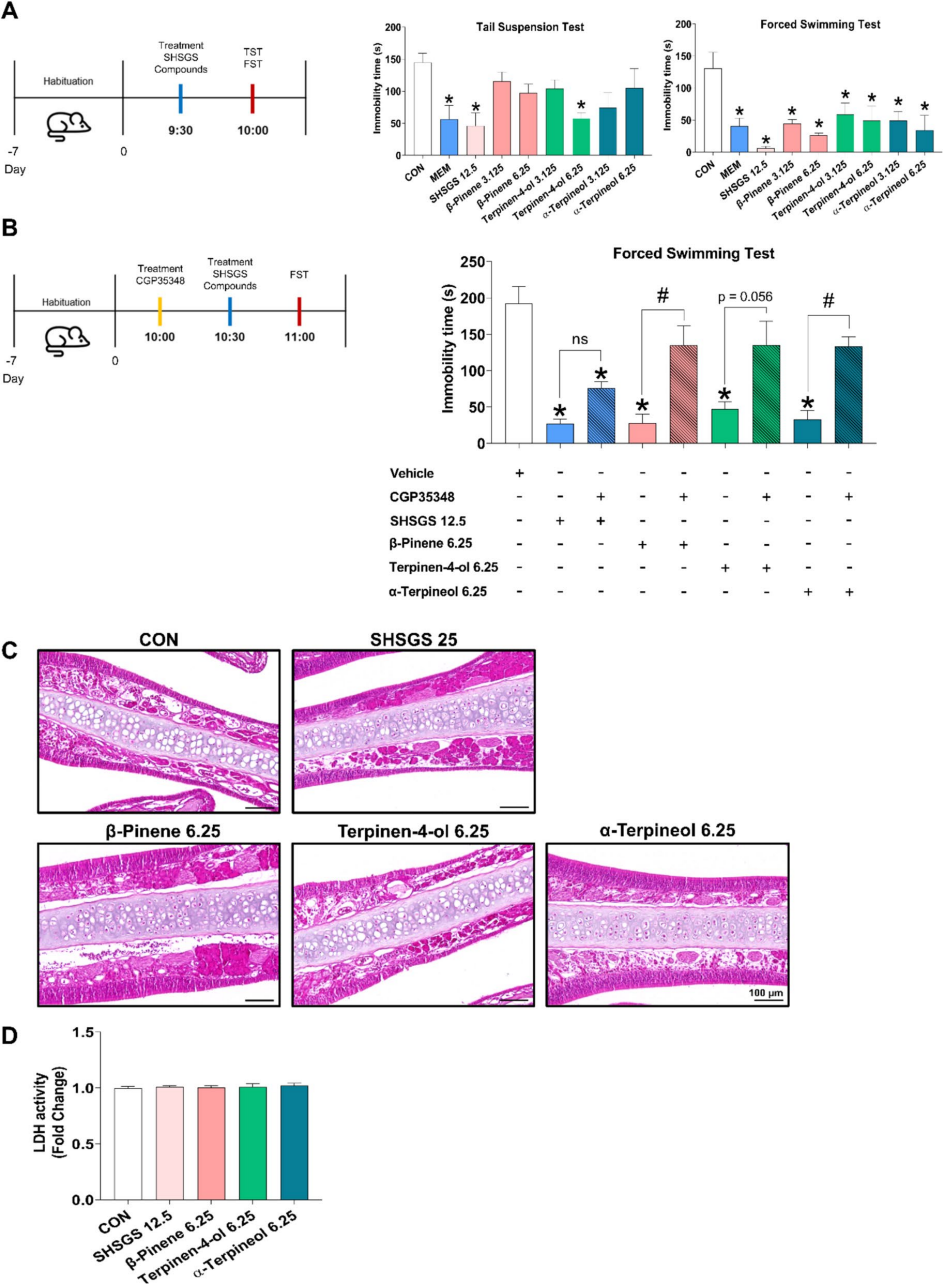
In vivo effects of SHSGS compounds. Effects of SHSGS compounds on mouse behavior in the **A** TST and FST. MEM (3.125 mg/kg) was the positive control. **B** Effects of SHSGS compounds under the presence of GABA_B1_ receptor antagonist CGP35348 in the FST. Data are presented as mean ± SEM (n = 5 per group). *p < 0.05 vs CON. **C** H&E staining results of the nasal mucosa after treatment with SHSGS compounds. Original magnification: 10X; scale bar: 100 μm, (n = 5 per group). **D** LDH activity in the olfactory bulbs under the treatment with SHSGS compounds. Data are presented as mean ± SEM (n = 5 per group)

In the TST, while all SHSGS compounds led to the reduction in immobility time, only terpinen-4-ol at 6.25 mg/kg showed the significant effect. In contrast, the FST showed that all SHSGS compounds significantly reduced immobility time compared to CON (Fig. 5A). Notably, under the administration of the GABA_B1_ receptor antagonist CGP 35348, the rapid antidepressant effects of all SHSGS compounds were blunted, with their immobility times even significantly higher than that of the single treatments (Fig. 5B). This supports the hypothesis that GABA_B1_ receptors play a critical role in the antidepressant-like effects of SHSGS compounds.

To evaluate the potential toxicity of SHSGS and its constituent compounds following single-dose administration, the highest dose used for each compound throughout the study was selected for assessment. Histological analysis of the nasal mucosa revealed that epithelial and submucosal structures remained intact across all treatment groups compared to the control. Although β-pinene treatment showed some moderate structural alterations, such as localized irritation or epithelial disorganization, no clear signs of toxicity were observed (Fig. 5C). Overall, tissue integrity was preserved in all groups. Similarly, the LDH activity assay in olfactory bulb tissues showed no significant differences between drug-treated groups and CON group (Fig. 5D).

#### α-Terpineol preferentially engages GABA_B1_ receptor signaling

**Fig. 6:**
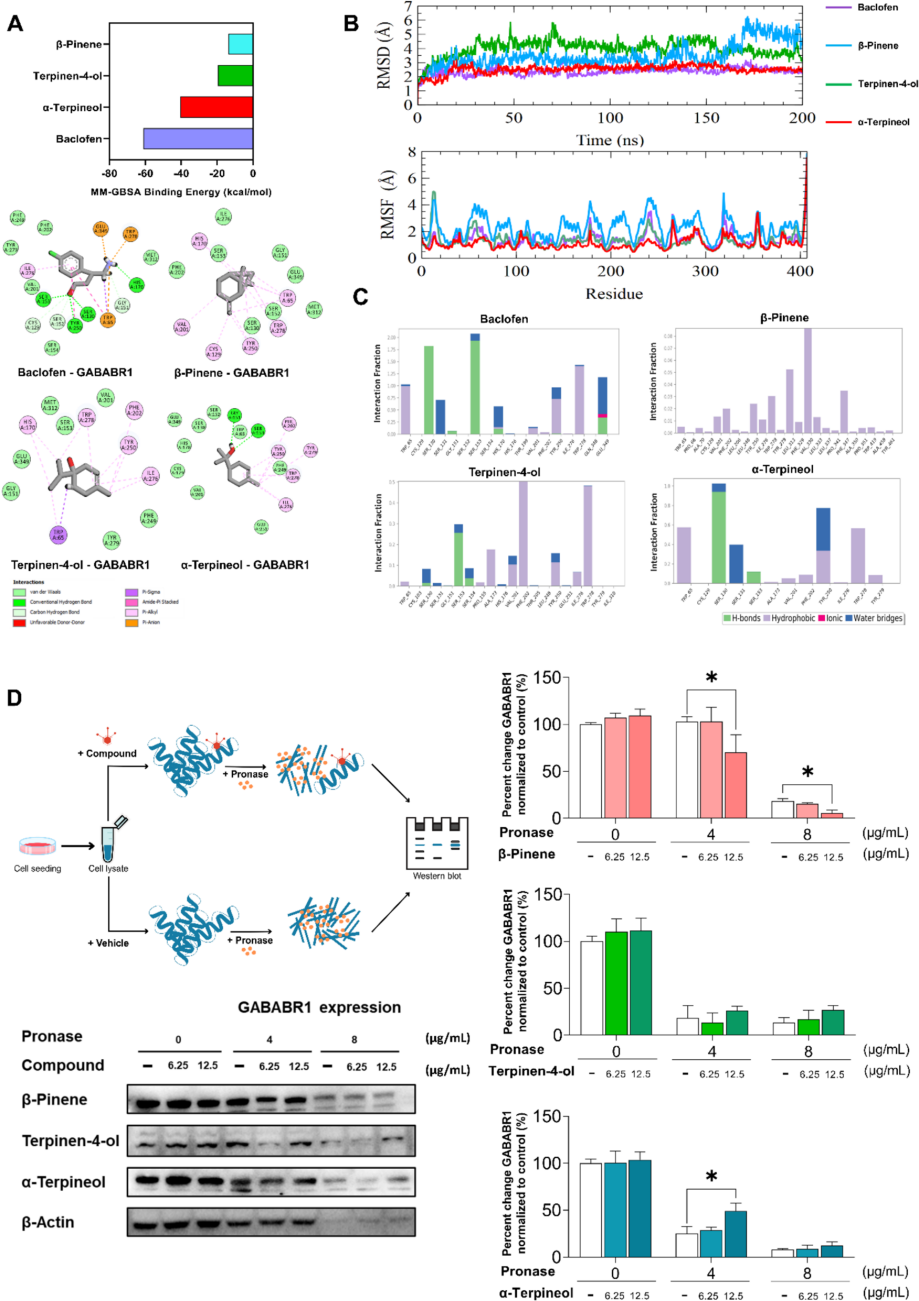
The interaction of SHSGS’s compounds towards GABA_B1_ receptors. **A** MM-GBSA binding energy (kcal/mol) and 2D docking modes. **B** Diagrams of protein root mean square deviation (RMSD) and root mean square fluctuations (RMSF). **C** Total protein–ligand contact analysis during 100 ns. **D** DARTS assay on SH-SY5Y cell line validating the binding between SHSGS compounds and GABA_B1_ receptor. Data are presented as mean ± SEM (n = 3 per group). *p < 0.05 vs CON

Molecular docking was used to predict the binding potentials of SHSGS compounds to GABABR1. As in Table 3 and Fig. 6A, except for the well-known GABAB receptor agonist baclofen with a higher binding affinity to GABABR1, α-terpineol exhibited strongest binding affinity (with docking score − 7.01 kcal/mol; MM-GBSA binding energy − 40.418 kcal/mol) among SHSGS compounds, followed by terpinen-4-ol (− 6.02 kcal/mol; − 19.474 kcal/mol) and β-pinene (− 3.75 kcal/mol; − 13.761 kcal/mol). In molecular simulation, α-terpineol exhibited a stable binding pattern, with RMSD values comparable to those of baclofen. In contrast, terpinen-4-ol displayed the highest RMSD values with significant fluctuations, indicating instability in its binding conformation. β-Pinene remained stable up to 150 ns but showed notable fluctuations thereafter, suggesting reduced binding stability in the later phase. α-Terpineol and terpinen-4-ol exhibited comparable residue stability and interactions within the binding site, as indicated by similar RMSF values. In contrast, β-pinene showed greater flexibility in binding site residues, potentially weakening its interaction stability (Fig. 6B). In total protein–ligand contact analysis, α-terpineol and terpinen-4-ol shared similar hydrogen bonds to the receptor with baclofen at Ser130 and Ser153, with terpinen-4-ol exhibiting slightly stronger interaction potential than α-terpineol. Conversely, β-pinene primarily formed hydrophobic interactions with Tyr250 and Trp278, suggesting a distinct binding mode (Fig. 6C).

**Table 3:** Docking scores of candidate Q-markers in SHSGS and potential targets

Compound	Docking score (kcal/mol)
	GABABR1
Baclofen	− 11.12
β-Pinene	− 3.75
Terpinen-4-ol	− 6.02
α-Terpineol	− 7.01

GABABR1, gamma aminobutyric acid type B receptor subunit 1

To validate the prediction from molecular docking, the DARTS assay was performed (Fig. 6D). Under the presence of pronase, α-terpineol demonstrated concentration-dependent protection of GABABR1 from pronase digestion, as evidenced by higher GABABR1 expression at 4 and 8 µg/mL pronase compared to the vehicle control. Notably, β-pinene treatment unexpectedly resulted in greater GABABR1 degradation compared to vehicle, suggesting a possible conformational modification or indirect interaction rather than a stabilizing binding event. Terpineol-4-ol showed no significant protective effect across conditions.

#### SHSGS candidate Q-markers enhance GABA_B1_ receptor and BDNF/TrkB/ERK signalings in primary hippocampal neurons

**Fig. 7:**
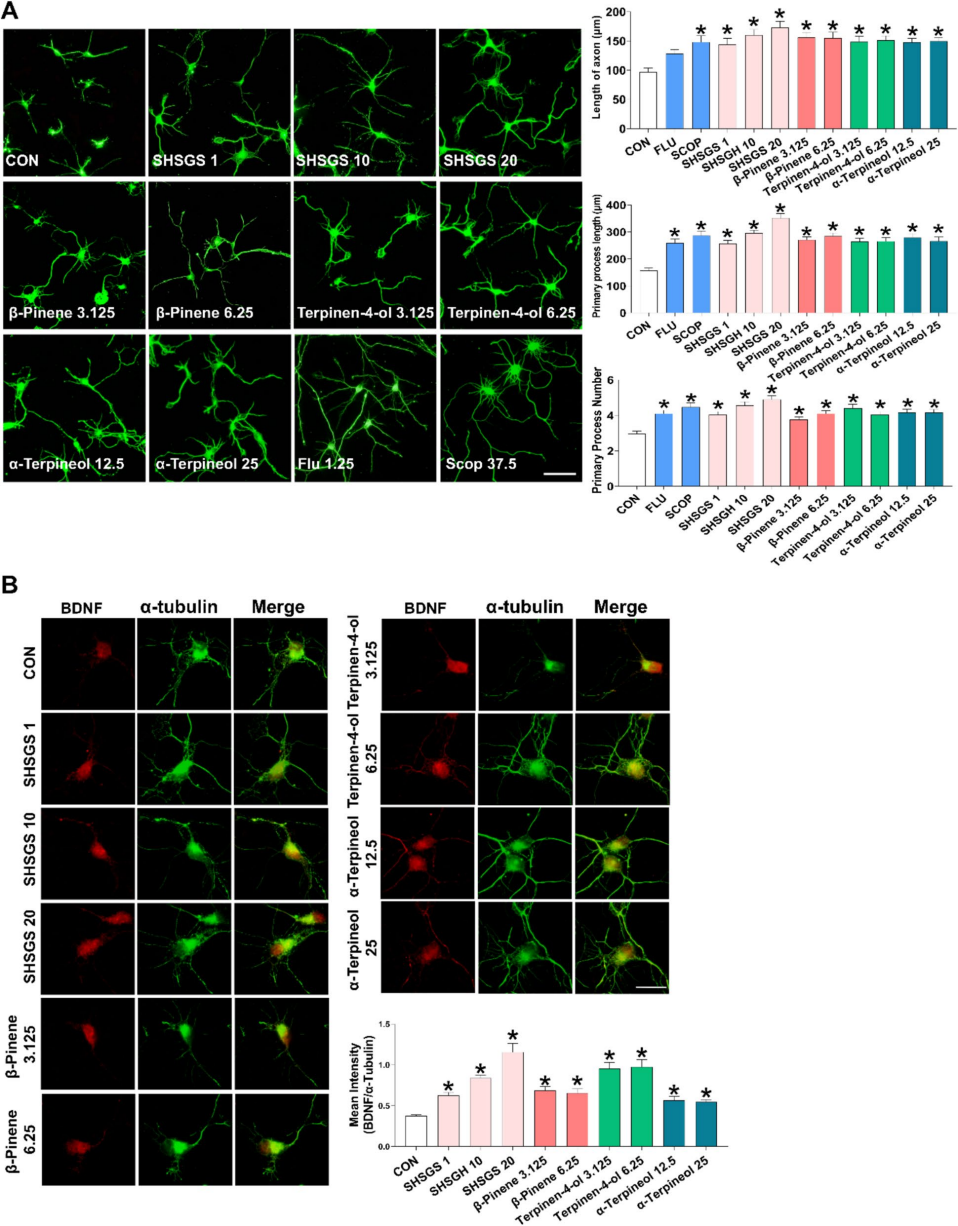

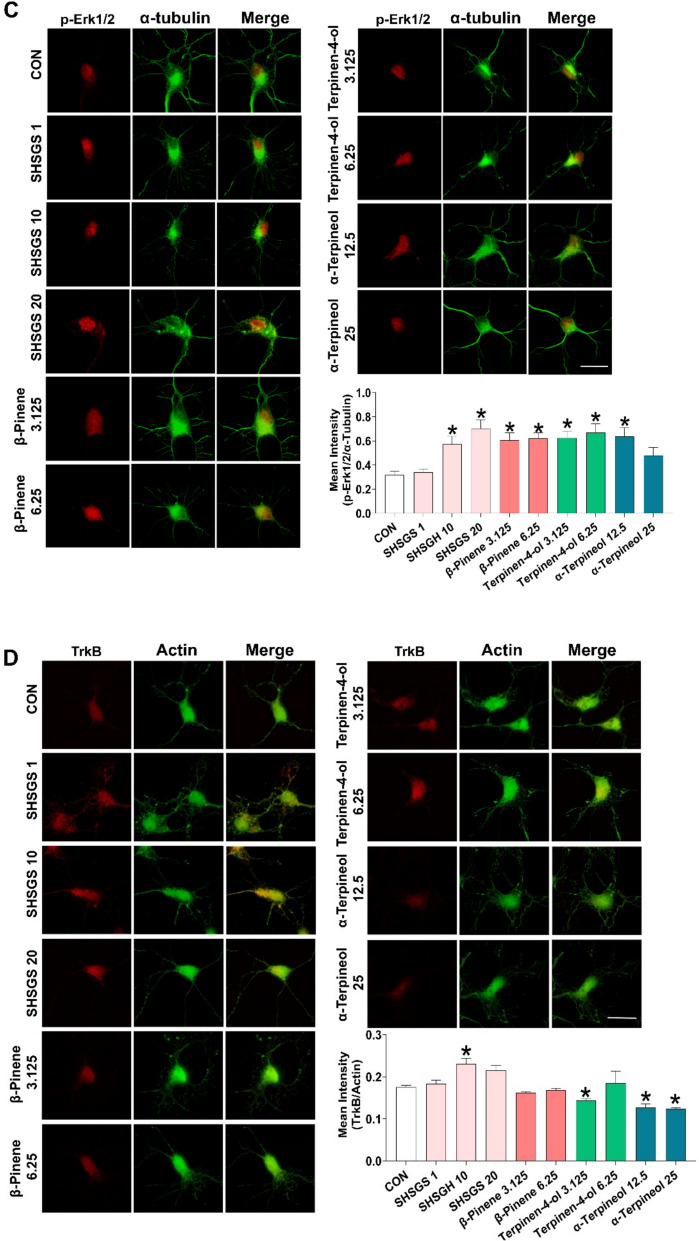

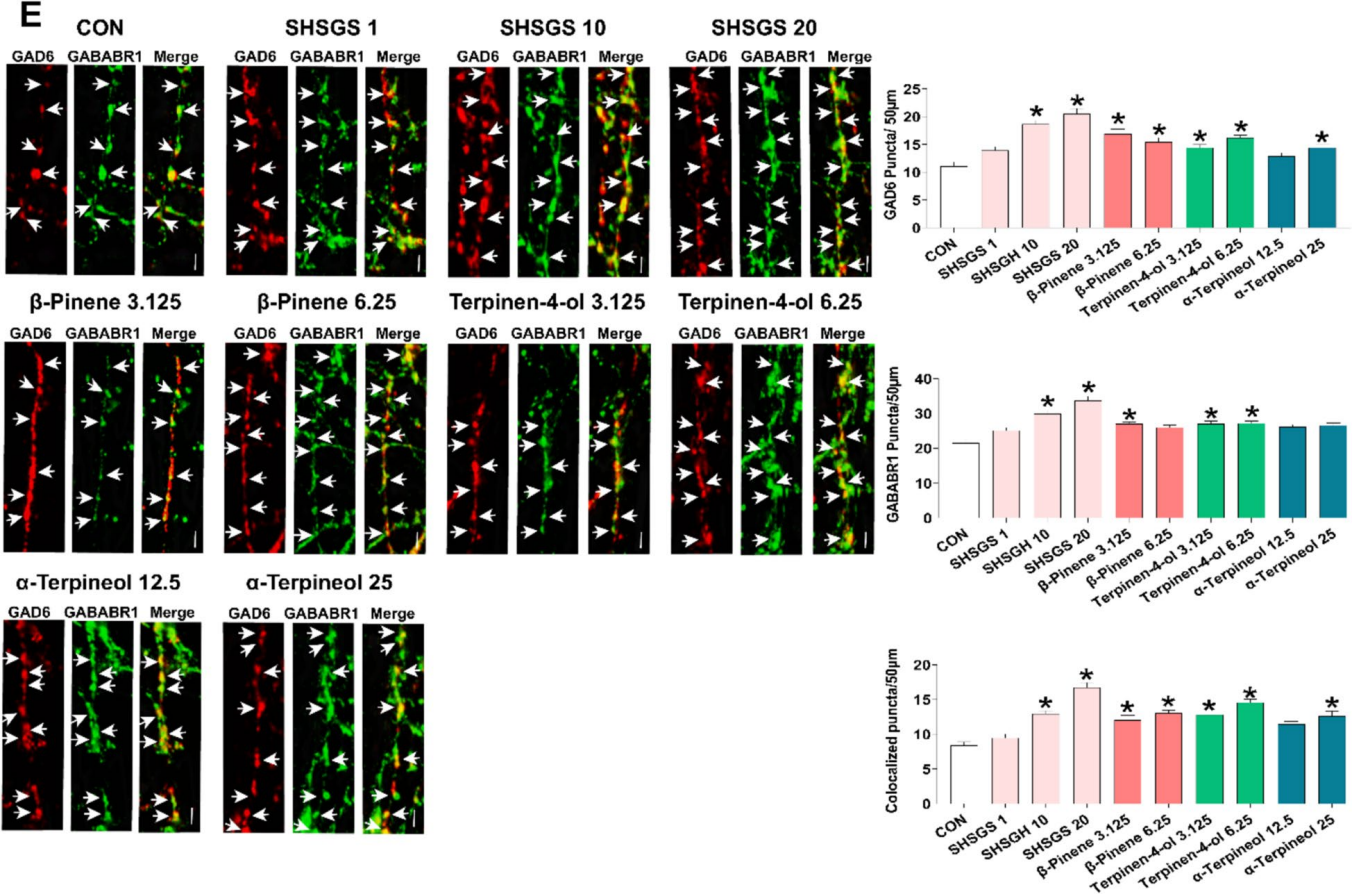
Effects of SHSGS compounds on hippocampal primary neurons. **A** Neuritogenic effect of SHSGS compounds on neurite outgrowth. Images showing representative fluorescent neurons on DIV3. Scale Bar: 50 μm. FLU (1.25 μM) and SCOP (37.5 μM) were the positive controls. Effect of SHSGS compounds on **B** BDNF, **C** p-Erk1/2, **D** TrkB expressions in red color with α-tubulin in green color. **E** Effect of SHSGS compounds on inhibitory synaptic development, with synaptic puncta in yellow, marked with co-localization of GAD6 in red and GABABR1 in green. Scale bar, 2 μm. Data are presented as mean ± SEM. *p < 0.05 vs CON

Embryonic-19 (E19) rat hippocampal neurons were treated with different concentrations of SHSGS and its compounds. Phase-contrast micrographs were acquired on DIV3 to determine the neurite outgrowth effect. SHSGS and its components promoted neuronal growth in a dose-dependent manner (Supplementary Fig. S1). SHSGS and three compounds significantly increased the total number and length of primary processes. Statistically, SHSGS increased all morphometric parameters at 1, 10, and 20 μg/mL. While the optimum doses for β-pinene were 3.125 and 6.25 μg/mL, those for terpinene-4-ol and α-terpineol were 3.125—6.25 μg/mL and 12.5—25 μg/mL, respectively (Fig. 7A). Hence, further analyses were conducted using only these effective doses. In addition, the neuritogenic effects of SHSGS and its components were compared with those of other acknowledged neuromodulatory agents, such as SCOP and FLU. All three components showed effects comparable to those of SCOP and FLU in promoting the number and length of the total processes, yet their effects were not as pronounced as those of SHSGS (Fig. 7A).

**Fig. 8:**
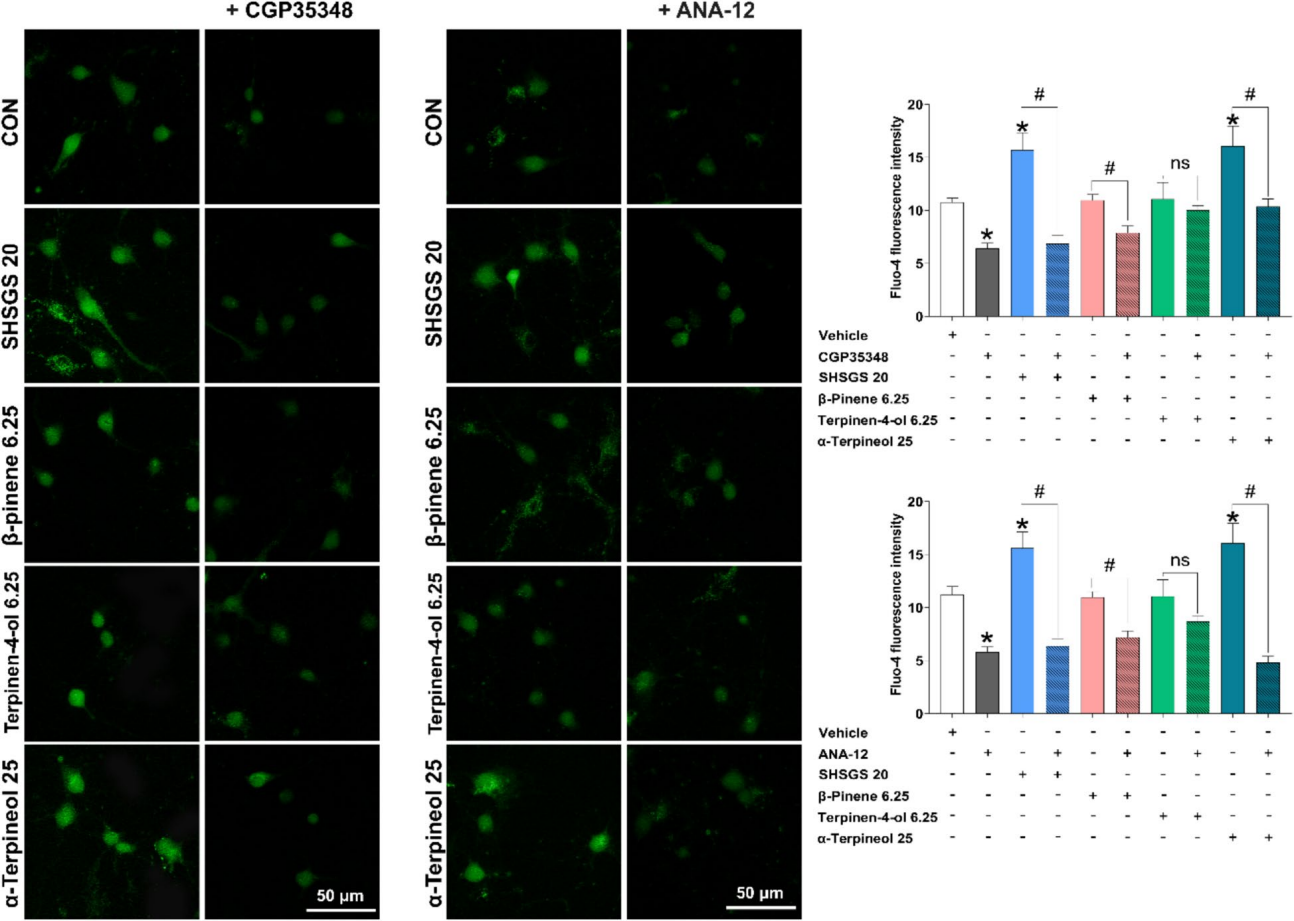
Measurement of intracellular calcium level on hippocampal primary neurons. Immunofluorescent images of SHSGS compounds-treated neurons under the presence of the TrkB receptor antagonist ANA-12 and GABA_B1_ receptor antagonist CGP35348, with quantitative analysis via the mean intensity of Fluo-4. Data are presented as mean ± SEM (n = 50 neurons per group). *p < 0.05 vs CON, #p < 0.05 vs SHSGS/compounds alone

Upon treatment with SHSGS and its compounds, higher expressions of BDNF and p-ERK were observed (Fig. 7B-C). TrkB expression was significantly increased by SHSGS at 10 μg/mL, terpinen-4-ol at 3.125 μg/mL, and α-terpineol at 12.5 and 25 μg/mL, while the others showed no significant effect (Fig. 7D). The effects of GABAergic signaling were investigated by examining GAD6 and GABABR1 expression. GAD6 expression was notably increased by SHSGS and the three compounds. GABABR1 expression was enhanced only by SHSGS at 10 and 20 μg/mL, β-pinene at 3.125 μg/mL, and terpinene-4-ol at 3.125 and 6.25 μg/mL (Fig. 7E). To determine the inhibitory synapse formation, the quantity of puncta that were co-localized were determined by counting the number within a certain length of 50 μm. SHSGS and its compounds promoted the colocalization of GABA-BR1 and GAD6 immunoreactivity (Fig. 7E). Fluo-4 calcium imaging revealed that SHSGS significantly elevated intracellular calcium levels in cultured hippocampal neurons, an effect that was reversed by co-treatment with the GABA_B1_ receptor antagonist CGP 35348 and the TrkB inhibitor ANA-12. Similarly, α-terpineol-induced calcium elevation was significantly attenuated by both inhibitors, suggesting involvement of the GABA_B1_ receptor–TrkB signaling pathway. In contrast, β-pinene and terpinene-4-ol did not produce significant changes in intracellular calcium levels under the same conditions (Fig. 8).

## Discussion

This study characterizes the rapid-onset antidepressant effects of SHSGS and delineates its molecular mechanisms through an integrated analysis of behavioral, cellular, and biochemical parameters. In contrast to conventional oral preparations of SHSGS requiring high doses (4–19.5 g/kg) and extended treatment durations (4–12 weeks), the present SDE-derived formulation was administered intranasally, and induced antidepressant effects at 12.5–25 mg/kg within 30 min [20, 21]. These effects were consistently observed across multiple behavioral analysis approaches, suggesting rapid pharmacodynamic engagement and central activity. Chemical profiling by GC–MS identified α-terpineol, β-pinene, and terpinen-4-ol as major volatile constituents of SHSGS. These monoterpenes exhibit physicochemical features favorable for CNS penetration, including low molecular weight (< 300 Da), moderate lipophilicity (logP 2–4), and non-ionic character [22]. In PAMPA-BBB assays, α-terpineol demonstrated significantly higher permeability than the reference compound diclofenac sodium, and all tested compounds showed moderate levels of permeability. These findings support the CNS accessibility of SHSGS constituents, probably facilitated by direct access to the CNS through the olfactory or trigeminal pathways while avoiding first-pass metabolism [23]. This also represents a paradigm shift from traditional water extracts, which primarily contain hydrophilic compounds, such as ferulic acid, naringin, and glycyrrhizic acid, with limited penetration of the CNS [24].

**Fig. 9:**
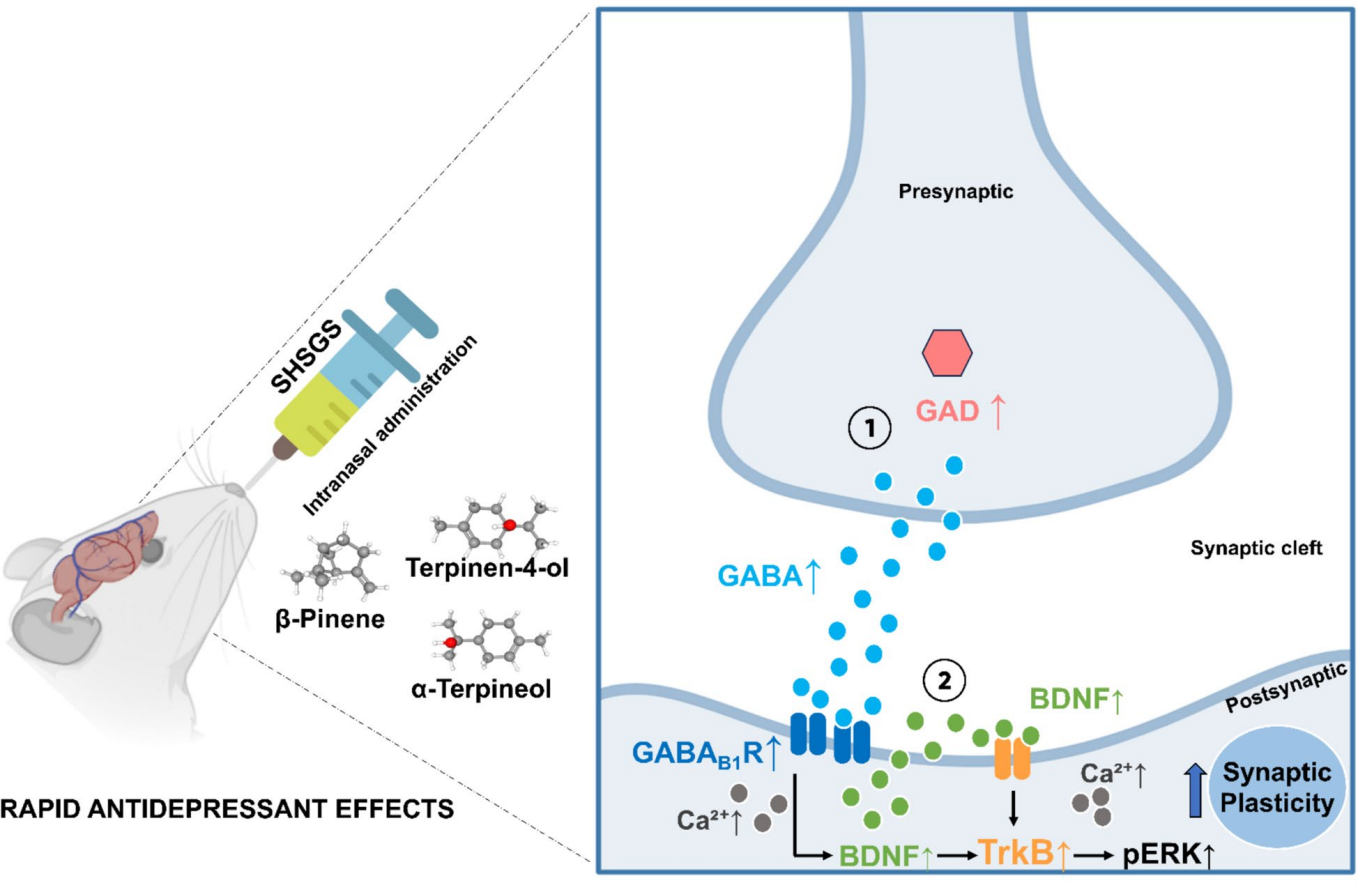
Schematic diagram of the mechanism underlying rapid antidepressant effects of intranasal SHSGS administration. **1** Following intranasal delivery, SHSGS enhances GAD expression in the presynaptic neurons and GABA release to the synaptic cleft. More GABA binding results in GABA_B1_ receptor activation, increased intracellular calcium, and downstream engagement of the BDNF/TrkB/ERK pathways. This cascade promotes synaptic plasticity. **2** Parallelly, SHSGS may also promote BDNF production in a GABA_B1_ receptor-independent mechanism, which then activates TrkB and downstream pathways, engaging synaptic plasticity and contributing to its overall antidepressant-like effects. Pink hexagon: glutamic acid decarboxylase enzyme (GAD); Light-blue circles: gamma-aminobutyric acid (GABA); Green circles: brain-derived neurotrophic factor (BDNF); Grey circles: calcium ions (Ca^2+^); Dark-blue rounded rectangle: gamma-aminobutyric acid type B receptor subunit 1 (GABA_B1_R); Orange rounded rectangles: Tropomyosin receptor kinase B (TrkB)

Mechanistically, SHSGS appears to enhance the expression of GAD in presynaptic neurons, thereby promoting GABA synthesis and increasing GABA release into the synaptic cleft. Increased GABA binding results in GABA_B1_ receptor activation, which in turn leads to intracellular calcium influx and subsequent activation of the BDNF/TrkB/ERK signaling cascade (Fig. 9). This pathway well aligns with a previous report, and is known to support synaptic plasticity and neuronal resilience [25]. Indeed, SHSGS was found to increase GABA levels, GAD and GABABR1 expressions in adult mouse hippocampal tissues and primary hippocampal neurons. These findings indicate robust activation of the GABAergic system and functional engagement GABA_B1_ receptors at the synaptic level. Notably, this mechanism aligns with previous evidence of diminished GABA levels, GAD67 expression, and GABA_B_ receptor function in patients with depression and in stress models [5]. Interestingly, the effects of SHSGS do not appear to be solely dependent on the GABAergic system, but rather involve concurrent activation of auxiliary pathways. While calcium imaging confirmed partial involvement of GABA_B1_ receptor, the antidepressant-like effect of SHSGS was not completely abolished by GABA_B1_ receptor antagonism, although a modest attenuation was observed. While calcium imaging confirmed partial involvement of GABA_B1_ receptor, these findings collectively suggest that SHSGS does not rely exclusively GABA_B1_ signalings. Instead, it likely engages additional GABA_B1_ receptor-independent mechanisms to enhance BDNF translation, which then activates TrkB/ERK signalings, as reported previously [26]. This broader engagement may underlie the retained efficacy of SHSGS even when GABA_B1_ signaling is pharmacologically blocked. However, it remains unclear whether BDNF/TrkB/ERK activation was indirectly mediated through GABA_B1_ receptor signaling or if SHSGS directly induced BDNF expression via an independent mechanism. In addition, co-administration of SHSGS with fluoxetine demonstrated enhanced rapid antidepressant effects. Our mechanistic studies revealed that SHSGS acts through GABAergic and BDNF/TrkB pathways, distinct from the serotonergic modulation of SSRIs, providing a biological basis for this therapeutic enhancement [27]. These findings suggest that SHSGS could serve as an adjunctive treatment to address the delayed onset associated with SSRIs.

Notably, our data reveal distinct mechanistic profiles for the three SHSGS candidate Q-markers. The antidepressant-like effect of α-terpineol was completely abrogated by GABA_B1_ receptor antagonism with CGP35348, and this compound robustly enhanced calcium influx in neurons, a functional readout of receptor activation. This, together with DARTS evidence showing that α-terpineol directly stabilizes GABABR1 against proteolytic degradation, strongly supports a model in which α-terpineol acts as a direct agonist or positive allosteric modulator of the GABA_B1_ receptor, with its antidepressant efficacy tightly linked to this target. β-pinene also required GABA_B1_ receptor signaling for its behavioral effects; however, it consistently did not function as GABA_B1_ receptor activator. Rather, β-pinene seemed to increase receptor susceptibility to proteolysis, suggesting a destabilizing or disruptive effect on GABABR1 conformation or its protein complex. This points to an indirect mechanism, where β-pinene’s antidepressant-like actions depend on the functional integrity of GABABR1, potentially by modulating its microenvironment or associated signaling pathways, but not through direct receptor engagement. Terpinen-4-ol, on the other hand, displayed a less definitive relationship with GABABR1 as its behavioral effect was not unequivocally blocked under GABA_B1_ receptor antagonism, and it neither induced calcium signaling nor conferred protection to GABABR1 under proteolysis. Terpinen-4-ol may influence GABAergic signaling weakly or likely engage additional pathways beyond the GABA_B1_ receptor. Although the SHSGS compounds exhibit distinct receptor interaction patterns, their consistent neuroprotective, anti-neuroinflammatory, and neuritogenic effects in vitro along with the rapid anti-depressant effects in vivo supports their potential in alleviating depression-related symptoms and biomarkers. Therefore, all three remain strong candidates as antidepressant Q-markers of SHSGS. This mechanistic diversity highlights the therapeutic value of multi-component herbal formulations and the importance of defining Q-markers not solely by abundance or pharmacological potency, but also by their mechanistic relevance within the therapeutic context.

Although our study provides strong evidence that intranasal SHSGS is a promising and rapid-acting antidepressant in both zebrafish and mouse models, several interspecies factors may affect the translation of preclinical findings into human therapeutics. First, rodents have a larger proportion of olfactory epithelium and more elaborate turbinates than humans, which can favor nose-to-brain transport. Mucociliary clearance rates and nasal enzymology also differ, potentially altering the residence time and metabolism of volatiles [28]. Second, BBB transporters and tight-junction profiles vary across species and brain regions. Compared to humans, mice have a higher overall expression and activity of P-glycoprotein, a crucial efflux transporter located in the BBB [29, 30]. This indicates that a compound that readily crosses the BBB in mice may be actively pumped out in humans. Furthermore, while core tight-junction proteins, such as claudin-5 and occludin, are conserved, their specific expression levels and regulatory mechanisms vary, contributing to differences in barrier integrity and function across species [31]. The pharmacokinetics of small monoterpenes are also influenced by volatility, pulmonary uptake, and first-pass metabolism; intranasal dosing circumvents the first-pass in rodents, but human exposure profiles may not match [32]. Third, the GABAergic circuitry, such as interneuron subtype distribution and GABAB receptor subunit expression, may differ between rodents and humans, leading to various interactions with the receptors [33]. To address translation, future work should quantify brain and plasma levels of α-terpineol, β-pinene, and terpinen-4-ol after intranasal dosing, and compare effects in human-derived neural systems such iPSC-derived neurons. Besides, allometric dose scaling and safety studies are necessary before clinical testing. This study is also limited by its focus on acute effects following a single administration, yet variables such as long-term efficacy, repeat-dosing safety, and pharmacokinetic stability were not addressed. In addition, only the GABAergic and BDNF/TrkB pathways were investigated, and other potentially relevant mechanisms were not explored. Broader transcriptomic or proteomic approaches may be useful for identifying additional signaling pathways involved in the SHSGS-mediated effects.

The concentrations of SHSGS and its compounds used in in vitro assays with immortalized cell lines may not mirror brain exposure after intranasal dosing and may respond differently to primary cells. We mitigated this by confirming the underlying mechanisms in primary hippocampal neurons and adult animal tissues, which showed strong consistency in activating the GABAergic and BDNF/TrkB/ERK pathways. Moreover, the efficacy of the candidate Q-markers was not only tested on immortalized and primary cell lines but also through changes in the phenotype of target receptor antagonist-treated mice. Although our PAMPA-BBB assay demonstrated the BBB permeability of the SHSGS compounds, in vivo evidence or pharmacokinetic profiling of the brain/plasma time courses of these compounds remains a subject for further study.

In addition to biological considerations, formulation stability remains a critical factor for intranasal delivery. As a volatile plant-based preparation, SHSGS may be prone to microbial contamination and chemical degradation over time. Lyophilization offers a practical approach for generating stable, reconstitutable dry powder formulations [34]. Moreover, advanced delivery technologies, such as mucoadhesive hydrogels and lipid-based nanoparticles, could enhance mucosal retention, improve permeability across the nasal epithelium, and protect active compounds from enzymatic breakdown [35, 36]. Future work should aim to optimize both the biological and pharmaceutical aspects of SHSGS to support its safe and effective clinical translation.

## Conclusion

This study demonstrated that intranasal SHSGS, optimized through SDE, achieved rapid anxiolytic and antidepressant effects within 30 min. Mechanistically, SHSGS appears to engage both GABAergic and BDNF/TrkB/ERK signaling pathways to promote synaptic plasticity and mood-related neuroadaptations. Among the key compounds, α-terpineol acts as a likely GABA_B1_ receptor agonist, β-pinene influences receptor stability indirectly, and terpinen-4-ol may act through alternative mechanisms. These mechanistic differences reinforce their roles as complementary Q-markers.

## Supplementary Information

Below is the link to the electronic supplementary material.

Supplementary file1 (DOCX 88 KB)

## Data Availability

The datasets used and/or analysed during the current study are available from the corresponding author on reasonable request.

## Abbreviations

BDNF: Brain-derived neurotrophic factor
CC: Closeness centrality
CNS: Central nervous system
CORT: Corticosterone
DC: Degree centrality
DIV: Days in vitro
EEG: Electroencephalogram
ERK: Extracellular signal-regulated protein kinase
FLU: Fluoxetine
GABA: Gamma-aminobutyric acid
GABABR1: GABA type B receptor subunit 1
GAD: Glutamic acid decarboxylase
GC–MS: Gas chromatography–mass spectrometry
GO: Gene Ontology
HPC: Hippocampus
IL-6: Interleukin 6
KEGG: Kyoto Encyclopedia of Genes and Genomes
LPS: Lipopolysaccharide
MEM: Memantine
NeuN: Neuronal nuclei
OFT: Open field test
PFC: Prefrontal cortex
SCOP: Scoparone
SHSGS: Sihosogansan
SDE: Simultaneous distillation–extraction
SSRI: Selective serotonin reuptake inhibitor
TCMSP: Traditional Chinese Medicine Systems Pharmacology Database
TNF-α: Tumor necrosis factor alpha
TrkB: Tropomyosin receptor kinase B
TST: Tail suspension test
