# Supplementary material for: Novel intranasal delivery of sihosogansan demonstrates rapid antidepressant activity via GABAergic and BDNF/TrkB pathways: identification of potential bioactive quality markers
Source: Chin Med. 2025 Oct 6;20:162. doi: 10.1186/s13020-025-01219-6 (PMC12498446; doi:10.1186/s13020-025-01219-6)
Supplement: Supplementary file 1 — Supplementary file1 (DOCX 88 KB) [file 13020_2025_1219_MOESM1_ESM.docx]

**Supplementary Materials**

**
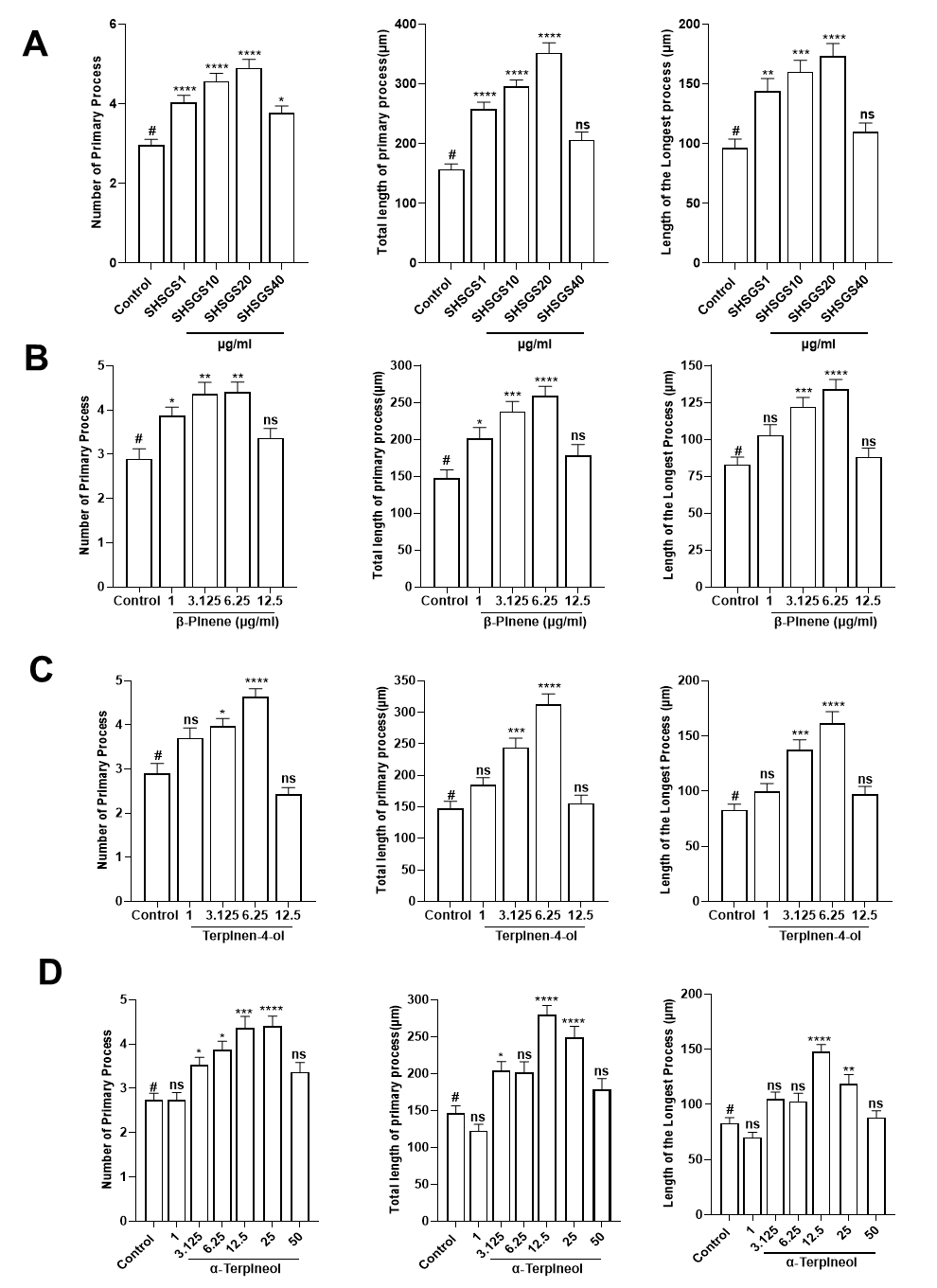
**

**Supplementary Figure S1.** Dose optimization of SHSGS, β-pinene, terpinen-4-ol, α-terpineol on neurite outgrowth. Neurite development of (A) SHSGS, (B) β-pinene, (C) terpinen-4-ol, and (D) α-terpineol via morphometric study describes the axon length, the number of primary processes, and their total length.  The significance of the statistical test was obtained by one-way ANOVA analysis (*P < 0.05, **P < 0.01, ***P < 0.001, ****P < 0.0001) compared to vehicle followed by a Tukey post hoc test. data dots represent the mean ± S.E.M. (standard error of the mean). ​

**Supplementary Table S1.** Instrument conditions for GC-MS analysis.

| **Item** | **Condition** |
| --- | --- |
| Detector | MSD |
| Column | DB-5MS (30 m x 250 μm, 0.25 μm thickness) |
| Oven temperature | 40°C (5 min) → 10°C/min, 280°C (10 min) |
| Helium gas flow (ml/min) | 1 |
| Injection volume (μL) | 1 |
| Split ratio | 1:20 |
| Inlet / detector temperature (°C) | 250 / 280 |
| Solvent delay (min) | 3 |
| Scan mode (amu) | 50-500 |

**Supplementary Table S2.** Target genes of SHSGS compounds.

| **Target gene** | **Protein** | **Related compounds** |
| --- | --- | --- |
| *ACHE* | Acetylcholinesterase | α-Terpinene, γ-Terpinene, Caryophyllene oxide |
| *ADH1A* | Alcohol dehydrogenase 1A | Methylcyclopentane, γ-Terpinene |
| *ADH1B* | Alcohol dehydrogenase 1B | Methylcyclopentane, D-Limonene, γ-Terpinene, β-Selinene |
| *ADH1B* | Alpha-1B adrenergic receptor | Methylcyclopentane, D-Limonene, γ-Terpinene, (1R)-(+)-Nopinone, Caryophyllene oxide |
| *ADH1C* | Alcohol dehydrogenase 1C | β-Myrcene, α-Terpinene |
| *ADRA1A* | Alpha-1A adrenergic receptor | (-)-β-Pinene, α-Terpinene, α-Terpineol, β-Elemene, β-Chamigrene |
| *ADRA1B* | Alpha-1B adrenergic receptor | (-)-β-Pinene, Terpinen-4-ol, α-Terpineol, β-Chamigrene |
| *ADRA1D* | Alpha-1D adrenergic receptor | Terpinen-4-ol |
| *ADRA2A* | Alpha-2A adrenergic receptor | α-Terpinene |
| *ADRA2C* | Alpha-2C adrenergic receptor | (-)-β-Pinene, α-Terpinene |
| *ADRB1* | Beta-1 adrenergic receptor | α-Terpinene, α-Terpineol |
| *ADRB2* | Beta-2 adrenergic receptor | α-Terpinene, Terpinen-4-ol, α-Terpineol |
| *AR* | Androgen receptor | α-Terpinene |
| *BCL2* | Apoptosis regulator Bcl-2 | β-Elemene |
| *CALM1* | Calmodulin | Terpinen-4-ol |
| *CCNB1* | G2/mitotic-specific cyclin-B1 | β-Elemene |
| *CDC2* | Cell division control protein 2 homolog | β-Elemene |
| *CDKN1A* | Cyclin-dependent kinase inhibitor 1 | β-Elemene |
| *CHRM1* | Muscarinic acetylcholine receptor M1 | (-)-β-Pinene, α-Terpinene, D-Limonene, Linalool, Terpinen-4-ol, α-Terpineol, β-Elemene, β-Selinene, β-Chamigrene, (+)-δ-Cadinene, Spathulenol, Caryophyllene oxide |
| *CHRM2* | Muscarinic acetylcholine receptor M2 | β-Phellandrene, (-)-β-Pinene, α-Terpinene, D-Limonene, Linalool, Terpinen-4-ol, α-Terpineol, (-)-Verbenone, β-Elemene, β-Selinene, β-Chamigrene, Spathulenol, Caryophyllene oxide |
| *CHRM3* | Muscarinic acetylcholine receptor M3 | β-Phellandrene, (-)-β-Pinene, Linalool, Terpinen-4-ol, α-Terpineol, (-)-Verbenone, β-Elemene, Cyperene, β-Selinene, β-Chamigrene, Spathulenol, Caryophyllene oxide |
| *CHRNA2* | Neuronal acetylcholine receptor subunit alpha-2 | α-Pinene, β-Phellandrene, (-)-β-Pinene, Terpinen-4-ol, β-Chamigrene |
| *CHRNA7* | Neuronal acetylcholine receptor protein, alpha-7 chain | β-Elemene, β-Chamigrene, α-Cyperone |
| *CTRB1* | Chymotrypsinogen B | α-Terpinene |
| *CYP101* | Cytochrome P450-cam | Methylcyclopentane, α-Pinene, (-)-β-Pinene, α-Terpinene, D-Limonene, γ-Terpinene, (1R)-(+)-Nopinone, Terpinen-4-ol, (-)-Verbenone, (+)-δ-Cadinene |
| *DPP4* | Dipeptidyl peptidase IV | α-Terpinene, γ-Terpinene, Caryophyllene oxide |
| *EIF6* | Eukaryotic translation initiation factor 6 | β-Elemene |
| *F10* | Coagulation factor Xa | Terpinen-4-ol |
| *F2* | Thrombin | α-Terpinene, Caryophyllene oxide |
| *GABRA1* | Gamma-aminobutyric acid receptor subunit alpha-1 | α-Pinene, β-Phellandrene, (-)-β-Pinene, β-Myrcene, α-Terpinene, D-Limonene, γ-Terpinene, Linalool, Terpinen-4-ol, α-Terpineol, (-)-Verbenone, β-Elemene, Cyperene, β-Selinene, β-Chamigrene, (+)-δ-Cadinene, Spathulenol, Caryophyllene oxide, Sedanolide, (E)-Ligustilide, α-Cyperone |
| *GABRA2* | Gamma-aminobutyric-acid receptor alpha-2 subunit | α-Pinene, (-)-β-Pinene, D-Limonene, Linalool, (1R)-(+)-Nopinone, Terpinen-4-ol, α-Terpineol, (-)-Verbenone, β-Elemene, β-Selinene, β-Chamigrene, Caryophyllene oxide, Sedanolide, α-Cyperone |
| *GABRA3* | Gamma-aminobutyric-acid receptor alpha-3 subunit | (-)-β-Pinene, D-Limonene, Linalool, Terpinen-4-ol, α-Terpineol, β-Elemene, β-Selinene, β-Chamigrene, Sedanolide, α-Cyperone |
| *GABRA4* | Gamma-aminobutyric-acid receptor subunit alpha-4 | β-Chamigrene |
| *GABRA5* | Gamma-aminobutyric-acid receptor alpha-5 subunit | α-Pinene, (-)-β-Pinene, D-Limonene, Linalool, Terpinen-4-ol, α-Terpineol, (-)-Verbenone, β-Elemene, β-Chamigrene, Sedanolide |
| *GABRA6* | Gamma-aminobutyric-acid receptor subunit alpha-6 | α-Pinene, (-)-β-Pinene, D-Limonene, Linalool, Terpinen-4-ol, α-Terpineol, β-Elemene, β-Selinene, β-Chamigrene, Caryophyllene oxide, Sedanolide |
| *GRM2* | Glutamate receptor 2 | Sedanolide |
| *HSP90AA1* | Heat shock protein HSP 90 | Terpinen-4-ol |
| *HTR2A* | 5-hydroxytryptamine 2A receptor | α-Terpineol |
| *HTR3A* | 5-hydroxytryptamine receptor 3A | Terpinen-4-ol |
| *IGHG1* | Ig gamma-1 chain C region | D-Limonene, Terpinen-4-ol, α-Terpineol |
| *KCNH2* | Potassium voltage-gated channel subfamily H member 2 | Terpinen-4-ol |
| *LTA4H* | Leukotriene A-4 hydrolase | α-Terpinene |
| *LYZ* | Lysozyme | Methylcyclopentane, (-)-β-Pinene, p-Cymene, (1R)-(+)-Nopinone |
| *MAOA* | Amine oxidase [flavin-containing] A | (-)-β-Pinene, α-Terpinene, α-Terpineol |
| *MAOB* | Amine oxidase [flavin-containing] B | α-Terpinene, α-Terpineol, β-Chamigrene, (+)-δ-Cadinene |
| *NCOA1* | Nuclear receptor coactivator 1 | D-Limonene, Terpinen-4-ol |
| *NCOA2* | Nuclear receptor coactivator 2 | (-)-β-Pinene, α-Terpinene, D-Limonene, Terpinen-4-ol, β-Elemene, Cyperene, β-Selinene, β-Chamigrene, (+)-δ-Cadinene |
| *NR3C1* | Glucocorticoid receptor | α-Terpinene |
| *NR3C2* | Mineralocorticoid receptor | Terpinen-4-ol, (-)-Verbenone |
| *PDE3A* | CGMP-inhibited 3',5'-cyclic phosphodiesterase A | Terpinen-4-ol, α-Terpineol |
| *PGR* | Progesterone receptor | Terpinen-4-ol, (-)-Verbenone |
| *PRKACA* | PKA Catalytic Subunit C-alpha | α-Pinene |
| *PRSS3* | Trypsin-3 | Methylcyclopentane |
| *PTGS1* | Prostaglandin G/H synthase 1 | (-)-β-Pinene, α-Terpinene, Terpinen-4-ol, β-Elemene, β-Selinene, β-Chamigrene, (+)-δ-Cadinene |
| *PTGS2* | Prostaglandin G/H synthase 2 | α-Pinene, (-)-β-Pinene, α-Terpinene, D-Limonene, γ-Terpinene, Terpinen-4-ol, β-Elemene, Cyperene, β-Selinene, β-Chamigrene, (+)-δ-Cadinene, Caryophyllene oxide, Sedanolide, (E)-Ligustilide |
| *RB1* | Retinoblastoma-associated protein | β-Elemene |
| *RHOA* | Transforming protein RhoA | β-Elemene |
| *RUNX1T1* | Protein CBFA2T1 | β-Elemene |
| *RXRA* | Retinoic acid receptor RXR-alpha | (-)-β-Pinene, α-Terpinene, Terpinen-4-ol, α-Terpineol, β-Elemene, β-Selinene, β-Chamigrene, (+)-δ-Cadinene |
| *SCN5A* | Sodium channel protein type 5 subunit alpha | Terpinen-4-ol |
| *SLC6A2* | Sodium-dependent noradrenaline transporter | (-)-β-Pinene, α-Terpinene, p-Cymene, Terpinen-4-ol, α-Terpineol, β-Elemene, β-Selinene, β-Chamigrene, (+)-δ-Cadinene, Sedanolide |
| *SLC6A3* | Sodium-dependent dopamine transporter | (-)-β-Pinene, α-Terpinene, α-Terpineol |
| *SLC6A4* | Sodium-dependent serotonin transporter | α-Terpineol |
| *TEP1* | Telomerase protein component 1 | β-Elemene |
| *TP53* | Cellular tumor antigen p53 | β-Elemene |

**Supplementary Table S3.** KEGG enrichment analysis.

| Pathway | **Related genes** |
| --- | --- |
| Neuroactive ligand-receptor interaction | *ADRA1A, ADRA2A, ADRA2C, ADRB1, ADRB2, CHRM2, CHRM3, CHRNA2, GABRA1, GABRA3, GABRA6, GRM2, HTR2A, NR3C1* |
| Serotonergic synapse | *HTR2A, HTR3A, MAOA, MAOB, PTGS1, PTGS2, SCL6A4* |
| Chemical carcinogenesis | *ADRB1, ADRB2, AR, BCL2, HSP90AA1, PGR, PTGS2* |
| Calcium signaling pathway | *ADRA1A, ADRB1, ADRB2, CALM1, CHRM2, CHRM3, HTR2A* |
| Taste transduction | *CHRM3, GABRA1, GABRA3, GABRA6, HTR3A* |
| cGMP-PKG signaling pathway | *ADRA1A, ADRA2A, ADRA2C, ADRB1, ADRB2, CALM1* |
| Salivary secretion | *ADRA1A, ADRB1, ADRB2, CALM1, CHRM3* |
| Cocaine addiction | *GRM2, MAOA, MAOB, SCL6A3* |
| Regulation of lipolysis in adipocytes | *ADRB1, ADRB2, PTGS1, PTGS2* |
| Adrenergic signaling in cardiomyocytes | *ADRA1A, ADRB1, ADRB2, BCL2, CALM1* |
| Amphetamine addiction | *CALM1, MAOA, MAOB, SCL6A3* |
| Prostate cancer | *AR, BCL2, HSP90AA1, TP53* |
| Pathways in cancer | *AR, BCL2, CALM1, HSP90AA1, PTGS2, RUNX1T1, TP53* |
| Tyrosine metabolism | *ADH1B, MAOA, MAOB* |
| Cholinergic synapse | *ACHE, BCL2, CHRM2, CHRM3* |
| Nicotine addiction | *GABRA1, GABRA3, GABRA6* |
| Parkinson disease | *CALM1, MAOA, MAOB, SCL6A3, TP53* |
| Dopaminergic synapse | *CALM1, MAOA, MAOB, SCL6A3* |
| Estrogen signaling pathway | *BCL2, CALM1, HSP90AA1, PGR* |
| Fluid shear stress and atherosclerosis | *BCL2, CALM1, HSP90AA1, TP53* |

**Supplementary Table S4.** GO enrichment analysis.

| **Subgroup** | **GO term** | **Related genes** |
| --- | --- | --- |
| Biological process | Chemical Synaptic Transmission | *CHRM2, CHRM3, CHRNA2, GABRA1, GABRA3, GABRA6, GRM2, HTR2A, HTR3A, SLC6A2* |
|  | Adenylate Cyclase-Activating Adrenergic Receptor Signaling Pathway | *ADRA2A, ADRA2C, ADRB1, ADRB2* |
|  | Adrenergic Receptor Signaling Pathway | *ADRA2A, ADRA2C, ADRB1, ADRB2* |
|  | Anterograde Trans-Synaptic Signaling | *CHRM2, CHRM3, CHRNA2, GRM2, HTR2A, HTR3A, SLC6A2* |
|  | Monoamine Transport | *SLC6A2, SLC6A3, SLC6A4* |
|  | Synaptic Transmission, GABAergic | *GABRA1, GABRA3, GABRA6* |
|  | Gamma-Aminobutyric Acid Signaling Pathway | *GABRA1, GABRA3, GABRA6* |
|  | Regulation Of Postsynaptic Membrane Potential | *GABRA1, GABRA3, GABRA6* |
|  | Positive Regulation Of Signaling Receptor Activity | *ADRA2A, ADRA2C, ADRB2* |
|  | Adenylate Cyclase-Activating G Protein-Coupled Receptor Signaling Pathway | *ADRA2A, ADRA2C, ADRB1, ADRB2* |
| Cellular component | Neuron Projection | *CHRM2, CHRM3, CHRNA2, GABRA1, GABRA3, GABRA6, GRM2, HTR2A, HTR3A, PTGS1, PTGS2, SLC6A2, SLC6A3, SLC6A4* |
|  | Dendrite | *CHRM2, CHRM3, CHRNA2, GABRA1, GABRA3, GABRA6, HTR2A* |
|  | Dendrite Membrane | *GABRA1, GABRA3, GABRA6* |
|  | Organelle Outer Membrane | *BCL2, MAOA, MAOB, PTGS2* |
|  | GABA-A Receptor Complex | *GABRA3, GABRA6* |
|  | Mitochondrial Outer Membrane | *BCL2, MAOA, MAOB* |
|  | Cation Channel Complex | *CALM1, HTR3A* |
|  | Neurotransmitter Receptor Complex | *HTR3A* |
|  | Flotillin Complex | *SLC6A3* |
|  | Astrocyte Projection | *GRM2* |
| Molecular function | Serotonin Receptor Activity | *CHRM2, CHRM3, HTR2A, HTR3A* |
|  | Estrogen Response Element Binding | *NR3C1, NR3C2, PGR* |
|  | Transmitter-Gated Monoatomic Ion Channel Activity | *GABRA1, GABRA3, GABRA6, HTR3A* |
|  | Monoamine Transmembrane Transporter Activity | *SLC6A2, SLC6A3, SLC6A4* |
|  | Sodium:Chloride Symporter Activity | *SLC6A2, SLC6A3, SLC6A4* |
|  | Ligand-Gated Monoatomic Anion Channel Activity | *GABRA1, GABRA3, GABRA6* |
|  | Acetylcholine Receptor Activity | *CHRM2, CHRM3, CHRNA2* |
|  | G Protein-Coupled Serotonin Receptor Activity | *CHRM2, CHRM3, HTR2A* |
|  | GABA Receptor Activity | *GABRA1, GABRA3, GABRA6* |
|  | G Protein-Coupled Amine Receptor Activity | *CHRM2, CHRM3, HTR2A* |
